# Functional reorganisation in chronic pain and neural correlates of pain sensitisation: A coordinate based meta-analysis of 266 cutaneous pain fMRI studies

**DOI:** 10.1016/j.neubiorev.2016.04.001

**Published:** 2016-09

**Authors:** Radu Tanasescu, William J. Cottam, Laura Condon, Christopher R. Tench, Dorothee P. Auer

**Affiliations:** a Clinical Neurology, Division of Clinical Neuroscience, University of Nottingham, Nottingham, UK; b Radiological Sciences, Division of Clinical Neuroscience, University of Nottingham, Nottingham, UK; c Arthritis Research UK Pain Centre, University of Nottingham, Nottingham, UK

**Keywords:** Chronic pain, fMRI, Coordinate based meta-analysis, Hyperalgesia, Neural pain signature

## Abstract

Maladaptive mechanisms of pain processing in chronic pain conditions (CP) are poorly understood. We used coordinate based meta-analysis of 266 fMRI pain studies to study functional brain reorganisation in CP and experimental models of hyperalgesia.

The pattern of nociceptive brain activation was similar in CP, hyperalgesia and normalgesia in controls. However, elevated likelihood of activation was detected in the left putamen, left frontal gyrus and right insula in CP comparing stimuli of the most painful vs. other site. Meta-analysis of contrast maps showed no difference between CP, controls, mood conditions. In contrast, experimental hyperalgesia induced stronger activation in the bilateral insula, left cingulate and right frontal gyrus.

Activation likelihood maps support a shared neural pain signature of cutaneous nociception in CP and controls. We also present a double dissociation between neural correlates of transient and persistent pain sensitisation with general increased activation intensity but unchanged pattern in experimental hyperalgesia and, by contrast, focally increased activation likelihood, but unchanged intensity, in CP when stimulated at the most painful body part.

## Highlights

⿢ Neural maladaptation in chronic pain conditions is poorly understood.
⿢ Large scale coordinate based *meta*-analysis of 266 cutaneous pain fMRI was performed.
⿢ Results support a shared neural pain response in chronic pain and healthy subjects.
⿢ Hyperalgesia leads to increased activation in an unchanged neural pattern.
⿢ Chronic pain patients show functional reorganisation depending on stimulation site.

## 1 Introduction

Chronic pain is a common public health concern. According to a report by Mantyselka et al. (2001), pain accounts for 40% of primary care consultations. A survey of adult Europeans found that the prevalence of moderate or severe chronic pain was 19% (Breivik et al., 2006), of whom 21% were diagnosed with depression related to their pain and 65% reported sleep disturbance due to pain (Breivik et al., 2006). Severe chronic pain was also found to be associated with increased 10 year mortality (Torrance et al., 2010). In the absence of more effective pain treatment personal suffering and socio-economic burden are huge, with the total annual cost of pain in the US estimated to be between $560 and $635 billion (Gaskin and Richard, 2012). This highlights the need for better understanding of the neural mechanisms underpinning the processing of chronic pain in order to begin addressing this issue.

Presently there is no clear temporal definition of when persistent pain is considered to be ⿿chronic⿿. However, there is consensus that chronic pain refers to pain persisting beyond its ecological alerting function, (i.e. when no benefit from healing can be assumed). As such chronic pain is considered a maladaptive state that is characterised not only by pain severity but also by a range of associated comorbidities including depression, distress and anxiety. A detailed taxonomy based upon symptom description and underlying etiology has been developed and recently updated by the International Association for the Study of Pain (www.iasp-pain.org/Taxonomy). Several etiological factors are coded, and in general nociceptive pain is distinguished from neuropathic pain: *nociceptive pain* involves activation of nociceptors through actual or threatened non-neural tissue damage, whereas *neuropathic pain* is defined as ⿿pain caused by a lesion or disease of the somatosensory nervous system⿿ (www.iasp-pain.org/Taxonomy).

The pathophysiology of chronic pain states is not well understood but there is a body of neurobiological and neuroimaging evidence suggesting that neuroplasticity is associated with the development of persisting pain (Dickenson, 2011, Henry et al., 2011, Tracey and Bushnell, 2009, Woolf, 2011). In animal models, several mechanisms of pain sensitisation were identified and characterised with peripheral sensitisation via activation of C-fibers, sodium channel alteration after nerve injury and central cord sensitisation due to increased spinal transmitter release and hyperexcitability of dorsal horn neurons (Dickenson, 2011). Modulation of stimulus-induced pain perception at the supraspinal level is highly complex with close interconnection of sensory, emotional and cognitive appraisal networks. Moreover, a key serotonergic descending facilitation pathway originates from the rostral brainstem and is believed to mediate fear and anxiety-related pain augmentation (Dickenson, 2011). Increased pain sensitivity (hyperalgesia) is well known in chronic pain patients (CP) resulting from a combination of peripheral and central sensitisation at the spinal cord level and supraspinal pain augmentation/facilitation (Woolf, 2011).

Central sensitization can be modelled in healthy human controls through priming, e.g. with intradermal capsaicin (active ingredient in chili, a TRPV1 receptor agonist), which leads to cross-modality hyperalgesia and allodynia (pain from non-noxious stimuli). The underlying neurophysiology is well characterised, and thought to reflect transient heterosynaptic changes making experimental central sensitisation a sound mechanistic model to test efficacy of centrally acting analgesics (Woolf, 2011). By contrast, top-down facilitatory processes are less well understood and robust experimental human models are lacking. Nevertheless, depression and low mood are commonly associated with chronic pain, and are thought to contribute to central pain augmentation and severity. Several studies have found that induction of depressed mood or sadness in healthy subjects increases pain sensitivity and augments pain unpleasantness (Berna et al., 2010, Pinerua-Shuhaibar et al., 2011, Villemure and Bushnell, 2009).

Modern functional neuroimaging has proved instrumental in gaining a deeper understanding of pain processing and its main modulatory factors in healthy volunteers (Baliki et al., 2007, Lee and Tracey, 2013) and chronic pain conditions. In line with the multidimensional nature of pain perception, experimental pain induces activation in several cortical and subcortical regions including primary (S1) and secondary (S2) somatosensory cortices, insula (INS), anterior cingulate cortex (ACC), thalamus, brainstem and prefrontal and parietal association areas (Melzack, 2001). These structures, often referred to as the ⿿pain matrix⿿ (Melzack, 2005), do not constitute a network that is unique to pain; in fact there are striking similarities with activation patterns of innocuous sensory stimuli (Iannetti and Mouraux, 2010). Consistent, but not pain specific, co-activation may relate to any of the aspects of the pain experience including saliency of stimuli (Iannetti et al., 2013), emotional responses (Hashmi et al., 2013) or action preparation (Perini et al., 2013), all of which could be modulated by chronic pain (Tracey and Mantyh, 2007). Functional neuroimaging might therefore be ideally suited to studying the functional reorganisation of both nociceptive and pain modulatory pathways in chronic pain syndromes, and to relate putative plasticity changes to etiologic pain subtypes and underlying mechanisms such as central sensitisation and negative affect.

Functional MRI (fMRI) studies have shown altered neural response to pain in CP compared to healthy controls (HC), for example (Baliki et al., 2006, Flor, 2003, Henderson et al., 2011, Napadow et al., 2010, Schweinhardt et al., 2006, Wrigley et al., 2009). However, there is some inconsistency between studies. Systematic reviews offer one method of exploring which of the reported altered neural responses are consistently observed, for example more frequent prefrontal, and less frequent ACC, S1, S2 and INS activation was noted in CP (Apkarian et al., 2005, Peyron et al., 2000). Recently coordinate based meta-analysis (CBMA) has become a popular alternative. CBMA analyses the results from related studies and performs statistical inference on them. Probably the most commonly used method computes an activation likelihood estimate (ALE) using the reported coordinates to identify and locate brain structures that are activated consistently across studies or differently between conditions/groups. In this way CBMA can mitigate low power and bias present in individual fMRI studies (Button et al., 2013, Wager et al., 2007). Several CBMAs of pain have been performed (Amanzio et al., 2013, Duerden and Albanese, 2013, Friebel et al., 2011, Lamm et al., 2011, Lanz et al., 2011, Simons et al., 2012). While some focused on general and modality-specific pain-induced changes in brain activity (Duerden and Albanese, 2013, Friebel et al., 2011, Lanz et al., 2011) others have studied the role of specific structures (Simons et al., 2012) or context-dependent effects (Amanzio et al., 2013, Lamm et al., 2011). CBMA has also been used to compare abnormal and altered pain states in CP or experimental hyperalgesia (Friebel et al., 2011, Lanz et al., 2011). Allodynia/hyperalgesia in chronic neuropathic and fibromyalgia patients was associated with increased activation likelihood in the left S2, ACC and right caudal anterior insula compared to experimental pain in HC (Friebel et al., 2011) while neuropathic pain decreased activation likelihood in most pain processing areas including S1, anterior and posterior insula and ACC compared to experimental hyperalgesia in HC (Lanz et al., 2011). There are, however, several methodological limitations of previous CBMA studies of pain. We have previously shown that the ALE methods are prone to false positive results (Tanasescu et al., 2015, Tench et al., 2013), and a new version of the software implementation (GingerALE, version 2.3.3) has been released to fix this. We have also shown that the false positive rate can increase with increasing study numbers (Tench et al., 2014); the current ALE algorithm does not correct for this. Furthermore, group comparisons make assumptions about the ALE that do not strictly hold (Duerden and Albanese, 2013, Lanz et al., 2011), or lack appropriate correction for the many statistical tests performed (Friebel et al., 2011). A further limitation is the uncontrolled heterogeneity between studies, often mixing functional neuroimaging methods (known to vary in sensitivity) or mixing a range of nociceptive receptor systems (superficial, deep and visceral).

To better understand the commonalities of altered pain processing in CP and any specific modulation related to its subtypes, and importantly to compare activation patterns in CP with experimental models of putative underlying mechanisms of pain sensitisation (hyperalgesia and low mood) in healthy controls, we undertook a CBMA on superficial pain fMRI studies and systematically addressed key experimental factors. We hypothesised that the neural response to experimental pain differs between CP and HC reflecting functional reorganisation, and that such changes can be partially modelled by experimental hyperalgesia or low mood in healthy controls. To overcome some of the limitations of previous pain CBMAs we employ a recently developed modified ALE based algorithm that reduces false positive results by using an intuitive type 1 error control scheme: the false cluster discovery rate (FCDR; similar to false discovery rate, but for clusters) control (Tench et al., 2013). We employ the same control for the contrast meta-analysis (CMA) used to locate differences in functional activation foci due to pain stimulus between groups (Tench et al., 2014). However, this test is sensitive only to strong local differences, so we also use an omnibus test that can detect subtle but distributed differences in the pattern when CMA is unrevealing (Tench et al., 2014). This test has also been used to explore differences due to experimental factors, such as pain stimulus modality (thermal, mechanical and electrical) to assess heterogeneity introduced by such factors. This, plus careful, and conservative study selection was used to create, as far as possible, homogeneous experimental groups. To this end, we limit the CBMA to fMRI studies and to cutaneous noxious stimuli thus eliminating unwanted heterogeneity from different imaging modalities and different nociceptive systems (superficial vs. deep and visceral).

## 2 Methods

### 2.1 Study inclusion and data selection

The literature search was performed according to the PRISMA guidelines for reporting meta-analyses and systematic reviews (Moher et al., 2009). Functional MRI studies of experimental cutaneous pain were searched both through standard literature databases (PubMed and Web of Knowledge) and from existing fMRI data repository NeuroSynth (neurosynth.org). NeuroSynth is a platform for large-scale, automated synthesis of fMRI data extracted from published articles (Yarkoni et al., 2011). We used the keywords (⿿MRI⿿ or ⿿magnetic resonance imaging⿿) AND (⿿functional⿿ or ⿿brain activation⿿ or ⿿neural activity⿿ or ⿿BOLD⿿) AND (⿿pain⿿ or ⿿noxious⿿ or ⿿nociception⿿ or ⿿hyperalgesia⿿ or ⿿allodynia⿿ or ⿿low mood⿿ or ⿿emotion⿿). In order to capture as many papers as possible, including studies on CP, additional searches have been performed using the strings (⿿MRI⿿ or ⿿magnetic resonance imaging⿿) AND (⿿functional⿿ or ⿿brain activation⿿ or ⿿neural activity⿿ or ⿿BOLD⿿) AND (⿿pain⿿ or ⿿noxious⿿ or ⿿nociception⿿ or ⿿hyperalgesia⿿ or ⿿allodynia⿿)AND (⿿low mood⿿ or ⿿emotion⿿), and (⿿MRI⿿ or ⿿magnetic resonance imaging⿿) AND (⿿functional⿿ or ⿿brain activation⿿ or ⿿neural activity⿿ or ⿿BOLD⿿) AND (⿿pain⿿ or ⿿noxious⿿ or ⿿nociception⿿) AND (⿿patients⿿ or ⿿neuropathic⿿ or ⿿chronic pain⿿ or ⿿hyperalgesia⿿ or ⿿allodynia⿿). For the search on NeuroSynth repository we used the key word ⿿pain⿿. Last accession of e-sources: 15 February 2015. The references of retrieved articles were then assessed for additional studies that could be considered for inclusion; along with the relevant references from review articles and meta-analyses. Abstracts were reviewed to select those studies involving noxious pain stimuli that had been induced via cutaneous stimulation. Studies reporting only activations for spontaneous pain in patients were excluded. Only studies that reported whole-brain group analysis as coordinates in the standard Talairach & Tournoux or Montreal Neurological Institute (MNI) reference space were considered (Evans et al., 1993, Talairach, 1988). Studies on HC with or without pain sensitisation were included. In cases where experimental pain was induced in addition to a cognitive manipulation task we only included the baseline coordinates of pain induction; if baseline coordinates were not given; the study was excluded from further analysis.

### 2.2 Data extraction and pre-processing

Activation coordinates were extracted from the papers manually and checked by three independent persons. Data extracted from each article were the authors⿿ names, date of publication, study population, sample size, stimulus modality, anatomical site of stimulation, laterality of bodily stimulation, brain activation coordinates and their associated standardised space (Talairach or MNI). Differences in the standardised coordinate space were addressed by converting all reported coordinates into Talairach space (Lancaster et al., 2007). We only included coordinates of activations since deactivations were reported by few studies, and the majority of studies did not comment on the presence or absence of deactivations.

Importantly, we also extracted activation coordinates from studies of between group comparisons allowing us to perform coordinate based meta-analysis of contrasts (MAC). Since such data is generated under controlled experimental conditions, MAC provides a powerful way to explore spatially consistent differences in activation intensity between the groups. This analysis is complementary to the contrast meta-analysis, which explores differences in consistency of reported structures, performed on similar groups.

Multiple studies reported in single papers can be strongly correlated. This would result in bias if these studies were considered independent (Turkeltaub et al., 2012). To prevent this, the extracted coordinates were merged into a single study if the paper reported results differing only by experimental factors such as different stimulus intensities, different body site stimulated, or data split by analysis e.g. late/early BOLD response. Studies using different subjects were not merged.

Different studies reporting the same results also violate the assumption of independence. Instances of repeated coordinates were identified and removed to prevent bias.

### 2.3 Coordinate based meta-analysis

Here we use a recently developed algorithm (LocalALE), which is freely available from http://www.nottingham.ac.uk/research/groups/clinicalneurology/neuroi.aspx. This algorithm utilizes the coordinates from fMRI studies to estimate where there is consistently reported activation across studies. It is based on the commonly used ALE algorithm (Turkeltaub et al., 2002) that utilizes a Gaussian, with specified full width half max (FWHM), model for each coordinate to estimate the activation likelihood. When many studies report coordinates in a similar location, the activation likelihood is high; a threshold for statistical significance is determined by a permutation test. LocalALE has important advantages over the more commonly used ALE algorithm: (1) the false positive rate is reduced, and (2) by adjusting the FWHM, heterogeneity between experiments that have different numbers of studies is reduced (Tench et al., 2013, Tench et al., 2014). Anatomical placement of significant clusters was reported in Talairach space using an automatic labelling scheme incorporated within LocalALE (Lancaster et al., 2000).

### 2.4 Contrast meta-analysis

Contrast meta-analysis is used to compare the reported activation foci between two groups, and has been described previously (Friebel et al., 2011). The results are clusters of coordinates where the activation pattern differs significantly between the groups. The null hypothesis is that the two groups report the same activation pattern. Permutation of the group variable is performed to estimate a *p*-value for each coordinate. This differs from the previously published scheme, where a *p*-value was estimated for each voxel; our scheme reduces the number of statistical tests performed by several orders of magnitude, and employs FCDR, to control type 1 errors (Tench et al., 2014).

### 2.5 An omnibus test of differences between two activation patterns

Contrast meta-analysis is sensitive to localised spatial differences in activation pattern between two groups. These differences need to be highly significant to survive correction for the multiple statistical tests performed. Consequently CMA is not sensitive to more subtle diffuse differences between the groups. Therefore, we have devised an omnibus test of difference in activation pattern between two groups (Tench et al., 2014).

The algorithm tests the combined *p*-values (sum, over coordinates, of the log *p*-values) generated by CMA using a method analogous to Fisher⿿s combined probability test; but without the assumption of independent, uniformly distributed, *p*-values. It achieves this by comparison with a null distribution generated from similarly combined *p*-values obtained by performing CMA on 1000 random permutations of the grouping variable. This test differs from CMA in that it requires not that single *p*-values be very small, but that the combined *p*-values be critical of the null hypothesis; this can occur when there is a diffuse pattern of subtle differences resulting in multiple small *p*-values, or a focal highly significant difference affecting just a few *p*-values.

### 2.6 Experimental procedure

#### 2.6.1 Study and data quality control

There is no consensus on minimum quality standards for, or how to quantify the quality of, functional MRI studies. Many fMRI studies are thought to be underpowered but there is no suitable formula based e.g. on number of subjects to determine a clear cut-off as the effect size for the chosen brain activation contrast will depend on the chosen activation and control task, respective presentation length and number of repeats in addition to technical factors such as field strength, sensitivity of head coil, acquisition protocol details and scanner-specific stability. Studies were thus considered eligible for inclusion when describing commonly used fMRI acquisition and analysis protocols.

Importantly, we used the diagnostic procedure detailed in (Tench et al., 2013) to check that studies appear commensurate in their respective groups. How commensurate each study is with all others was quantified by computing the mean activation likelihood across coordinates within the study, then comparing these to the distribution of mean activation likelihood values generated using an equal number of random coordinates; if the observed mean was in the high tail of this distribution, the study was considered commensurate. This test is sensitive to coordinate extraction errors and was used to pinpoint outlying studies to be scrutinised further and corrected as necessary. Further analysis was performed only after checking that the data are correct, and that the studies were included in the appropriate experimental group.

#### 2.6.2 Statistics

The CBMA, and MAC, were performed using an FCDR of 0.05, so of the significant clusters identified at most 5% would be expected under the null hypothesis. Contrast meta-analysis is less sensitive and performed using an FCDR of 0.1. For primary analyses (HC vs. CP) trends towards significance are also noted. The omnibus test of differences was considered significant if *p* ⿤ 0.05.

#### 2.6.3 Comparing activation pattern between groups

Comparisons of study groups were performed using three types of analysis. Plotting the frequency with which the studies report activations in each Talairach structure visually highlights where the groups differ, and where they are similar; structures were determined as the nearest grey matter Talairach structure to each reported coordinate. Comparison of groups by CMA revealed clusters of coordinates in locations where the activation patterns differ statistically. If this was unrevealing, the omnibus test of differences was used to more sensitively detect any differences, but without revealing where those differences are.

### 2.7 Experimental factors affecting the neural pain response pattern

To maximise statistical power experimental groups were created to contain as many studies as possible. This involved merging subgroups differing by experimental factors that might affect the functional response. Such differences would be a source of heterogeneity. The omnibus test was used here to explore such sources of heterogeneity.

It has been suggested that the encoding of painful stimuli takes place primarily in the contralateral hemisphere to the stimulation site (Coghill et al., 1999). Mirroring the coordinates from left-sided body stimulation about the y-axis (multiplying the x coordinate by ⿿1) would then maintain homogeneity across all studies, as long as the hemispheric lateralization is of minor importance in pain processing. Such mirroring has been used in a previous CBMA of pain (Lanz et al., 2011), but has recently been challenged (Duerden and Albanese, 2013). We compared subgroups of HC, where the grouping was right sided stimulation (subgroup RIGHT_HC_, Table 1) and left sided stimulation (subgroup LEFT_HC_, Table 1). The analysis was performed twice, once with original coordinates, and once with the coordinates mirrored in the left sided stimulus group. The results were used to determine whether mirroring of coordinates was performed throughout all subsequent experiments.

**Table 1:** Study groups included in the CBMA (in grey: main groups; in white: sub-groups).

Group	Detail	Population	Papers	Studies Extracted	Study numbers after merging	Coordinates extracted	Subjects
HC	All HC	HC	154	180	155	2780	2278
MECH_HC_	Mechanical	HC	24	28	25	391	325
THERM_HC_	Thermal	HC	104	120	104	1855	1520
ELEC_HC_	Electrical	HC	28	33	30	534	453
RIGHT_HC_	Right Stimulus	HC	64	75	65	1200	951
LEFT_HC_	Left Stimulus	HC	77	85	79	1249	1176
REST_HC_	Pain vs. rest	HC	76	91	78	1296	1128
INNOC_HC_	Pain vs. innocuous	HC	66	75	67	1216	912
CUED_HC_	Cued	HC	33	36	34	577	587
NCUED_HC_	Non-cued	HC	121	145	123	2199	1691
CP	All CP	CP	32	38	32	514	506
NEUR_CP_	Neuropathic	CPP	16	21	16	322	177
MSK_CP_	MSK	CPP	8	9	8	125	122
FM_CP_	FM	CPP	8	9	8	81	207
CS_CP_	Clinical Site	CPP	16	19	16	321	192
OS_CP_	Remote Site	CPP	16	19	16	193	309
OS-FM_CP_	As OS but excluding all FM studies	CPP	8	11	8	126	102
ALDN_CP_	Allodynic	CPP	11	12	11	199	143
NOX_CP_	Noxious	CPP	23	27	23	333	400
MECH_CP_	Mechanical CPP	CPP	20	21	20	289	381
HYPER_HC_	Hyperalgesia	HC	9	11	11	188	116

Subgroups of HC studies by modality (electrical, mechanical, and thermal) were formed (subgroups MECH_HC_, THERM_HC_, ELEC_HC_; Table 1). HC studies were also grouped by baseline stimulus: ⿿pain versus (vs.) innocuous stimuli⿿ and ⿿pain vs. rest⿿ (subgroups INNOC_HC_ and REST_HC_; Table 1), and by ⿿cued⿿ and ⿿non-cued⿿ stimulation (subgroups CUED_HC_ and NCUED_HC_; Table 1). Relevant subgroups were compared.

Pain sensitisation in CP may be affected by site and type (noxious or allodynic) of stimulus. In the CP group, half of the studies applied a stimulus at the most painful clinical site (CS) of the chronic pain (CS_CP_ subgroup; Table 1), while half used other bodily sites (OS) stimulation (OS_CP_ subgroup; Table 1); because all fibromyalgia (FM) studies included nociceptive stimulation remotely from any area reported by participants as being chronically painful (finger/thumb stimulation⿿6 studies; hand stimulation⿿2 studies), we included the eight FM studies in the OS_CP_ subgroup. To assess the influence of FM studies on the results, comparison between CS and OS was also performed excluding FM from the OS group (using the omnibus test). Around one quarter of the CP studies used non-noxious stimuli at the allodynic site (ALDN_CP_ subgroup; Table 1) whilst the rest used noxious painful stimuli (NOX_CP_ subgroup; Table 1). These groups were compared by omnibus test. Moreover, we explored the effect of matching pain intensity between groups or conditions based on perceived pain intensity of stimulus intensity. We found that approximately half of the reported comparisons of CP vs. HC and hyperalgesia vs. normalgesia (MAC⿿s) used standardised stimulus intensity (stimulus-matched paradigm) with the other half of studies adapting stimulus intensity to standardise perceived pain intensity (pain percept-matched paradigm). Due to the small sample sizes and potential inhomogeneity between these experiments, they were compared for differences using the omnibus test.

### 2.8 Neural pain response pattern in CP and HC

Coordinate based meta-analysis was used to find clusters of coordinates due to painful stimuli in HC and CP groups. The nearest Talairach structure to the centre of these clusters is reported, and considered to be of functional relevance to pain processing. The frequency with which each Talairach structure was reported was evaluated.

### 2.9 Differences in the pain response pattern between CP and HC

The neural signature of pain in CP and HC subgroups were compared using CMA to explore differences in activation patterns, and using MAC to explore differences in activation intensity. It is expected that the likelihood of activation shows significant spatial differences, reflecting functional reorganisation as previously suggested (Baliki et al., 2006, Flor, 2003, Henderson et al., 2011, Lanz et al., 2011, Napadow et al., 2010, Schweinhardt et al., 2006, Wrigley et al., 2009). We furthermore expected MAC differences related to hyperalgesia in CP.

#### 2.9.1 Is functional reorganisation in CP limited to neuropathic pain?

CP studies were sub-grouped by etiology (neuropathic (NEUR_CP_; Table 1), nociceptive musculo-skeletal (MSK_CP_; Table 1), and fibromyalgia (FM_CP_; Table 1). There are distinct neurophysiological differences between neuropathic and musculo-skeletal pain disorders with controversies regarding the classification of FM_CP_ (Phillips and Clauw, 2013, Rowbotham, 2005, Schnitzler and Ploner, 2000). Thus, there may be differences in the functional response to pain stimulation between these groups with the expectation that neuropathic pain is characterised by more prominent functional reorganisation. Relevant comparisons were made between these subgroups using CMA and the omnibus test of differences between groups.

#### 2.9.2 Can functional reorganisation in CP be modelled by hyperalgesia in HC?

To assess whether functional reorganisation in CP is related to peripheral and central sensitisation as putative mechanisms, we studied the activation pattern specific to experimental hyperalgesia in healthy controls as a model of pain sensitisation. To this end we studied the pattern of pain processing in HC after experimental induction of hyperalgesia (HYPER_HC_ subgroup; Table 1) in comparison with normalgesia by CBMA of the hyperalgesia group, and by CMA and MAC between hyperalgesia and normalgesia. The frequency of reporting brain structures activated in each group was also explored. We expected significantly increased likelihood and increased intensity of activation due to central sensitisation in hyperalgesia (Woolf, 2011).

#### 2.9.3 Can functional reorganisation in CP be modelled by low mood in HC?

To assess whether functional reorganisation in CP can be modelled by emotional status during painful stimulation, we studied the activation pattern specific to low mood in healthy controls under painful stimulation. Due to incomplete study reporting, we were unable to perform CMA between normal and low mood. Instead, we performed MAC of studies comparing painful stimulation with or without concomitant low mood condition (LM_HC_ subgroup; Table 1). We expected to detect increase in activation intensity during the low mood condition in emotional circuits and structures contributing to central pain augmentation and chronification.

## 3 Results

**Fig. 1:**
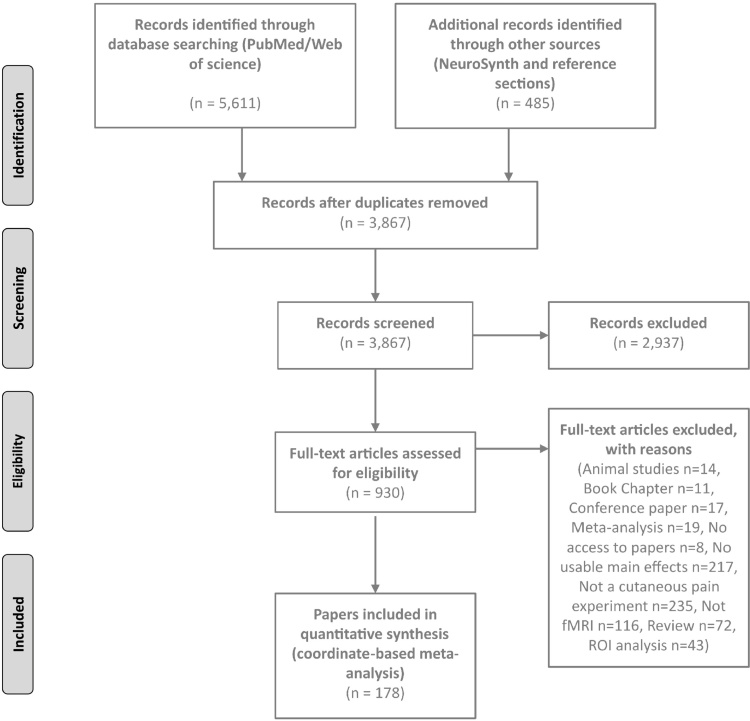
Flowchart of fMRI papers included in the analysis.

A total of 3815 reported coordinates of activation foci were extracted from 178 papers reporting on 266 fMRI studies of cutaneous experimental pain in 3014 subjects: 180 studies involved HC (2278 subjects; median 12, range 4⿿61 per study), and 38 studies involved CP (506 subjects; median 12, range 5⿿83 per study) (see Table S1 in Supplementary Material for included studies and Fig. 1). Coordinates from experimental hyperalgesia in HC and CP (11 studies, 116 subjects; 3 studies, 26 subjects) and pain studies in HC involving low mood induction (5 studies, 114 subjects) were also extracted.

The studies were divided into modality and condition-specific sub datasets (Table 1). Relevant contrast data from 37 studies suitable for MAC were available for extraction from 34 papers (Table 2).

**Table 2:** Study groups included in the coordinate based meta-analysis of contrasts (MAC), consisting of coordinates from fMRI studies of between-group comparisons.

Group	Detail	Population	Papers	Studies extracted	Study numbers after merging	Coordinates extracted	Subjects (no.)
CP_HC_MAC_	CP > HC activation intensity	CP, HC	17	18	17	103	221
HYPER_HC_MAC_	Hyperalgesia > normalgesia activation intensity	HC	9	11	9	146	121
HYPER_CP_MAC_	Hyperalgesia > normalgesia activation intensity in chronic pain patients	CP	3	3	3	38	26
LM_HC_MAC_	Low mood	HC	5	5	5	46	114

Chronic pain patient conditions included neuropathic pain syndromes (chronic regional pain syndrome, n = 4, trigeminal neuralgia, n = 1; burning mouth disorder, n = 1; syringomyelia, n = 1; post-herpetic neuralgia, n = 2; peripheral neuropathy n = 3; headache, n = 3; vulvar vestibulitis syndrome, n = 1); primary nociceptive musculo-skeletal disorders (low back pain, n = 6; osteoarthritis, n = 2) and fibromyalgia (FM, n = 8). We additionally subdivided CP studies according to the site of the nociceptive stimulation: at the most painful clinically affected site (CS_CP_; Table 1), or at another body site (OS_CP_; Table 1).

### 3.1 Experimental factors affecting the neural pain response pattern

Subgroups of HC studies using right sided pain stimuli (RIGHT_HC_; Table 1) and left sided pain stimuli (LEFT_HC_; Table 1) were compared using the omnibus test, with and without mirroring the coordinates in the latter group. The difference was significant when comparing the original coordinates (*p* = 0.036), but much more so when comparison was done using mirrored coordinates from the left sided stimulus studies (*p* = 0.002) demonstrating highly significant lateralisation of neural pain processing that cannot be neglected. Consequently, all subsequent experiments used original coordinates only.

The three different modalities of pain stimulation (mechanical, electrical, thermal) showed largely overlapping brain activation patterns (data not shown). However, the omnibus test did suggest some differences (mechanical vs. thermal: *p* = 0.02; mechanical vs. electrical: *p* = 0.04; electrical vs. thermal: *p* = 0.16).

We compared HC studies contrasting pain against rest (subgroup REST_HC_; Table 1) to studies contrasting pain against innocuous sensory stimuli (subgroup INNOC_HC_; Table 1). No differences were detected (*p* = 0.6).

We also compared studies employing cued painful stimulation (subgroup CUED_HC_, Table 1) to non-cued painful stimulation (subgroup NCUED_HC_, Table 1). Again, no differences were observed (*p* = 0.13).

We compared the CP subgroup in which nociceptive stimulus was applied to the site of maximal clinical pain (CS_CP_) to the CP subgroup in which painful stimulus was applied remotely to that site (OS_CP_). The OS_CP_ subgroup included all eight FM studies, as none used stimulation of most painful body sites. The stimulated body parts in the two subgroups were: upper limb (CS_CP_ 5 studies, OS_CP_ 13 studies), lower limb (CS_CP_ 6 studies, OS_CP_ 1 study), trunk (3 studies⿿all CS_CP_), and head and face (3 studies in each group). The omnibus test suggested some difference in activation pattern between these groups (*p* = 0.006). We also compared the CS_CP_ group with an OS_CP_ group that excluded FM (OS-FM_CP_). The omnibus test still indicated a difference in activation patterns between these groups (*p* = 0.03).

The design differences in matching CP and controls for pain intensity were unlikely to have masked true group differences as a comparison by omnibus test of perception versus stimulus intensity matched studies revealed no difference (*p* = 0.96). The same was found for hyperalgesia vs. normalgesia contrasts revealing no significantly difference when comparing perception and stimulus matched studies using the omnibus test (*p* = 0.6).

We compared the CP subgroup employing allodynic pain stimulus to the subgroup employing noxious stimuli; allodynic (subgroup ALDN_CP_; Table 1) vs. noxious (subgroup NOX_CP_; Table 1). However, we detected no significant differences in the activation likelihood by omnibus test (*p* = 0.46).

Aiming for large groups to increase power, subsequent experiments included studies using electrical, thermal, and mechanical modality types, rest and innocuous contrast conditions, cued and non-cued painful stimulus, and perception and stimulus intensity matched studies. However, the differences by pain stimulation modality, and by site of stimulation in the CP group, may be a source of heterogeneity.

### 3.2 The neural pain response pattern in chronic pain patients and in healthy controls

**Fig. 2:**
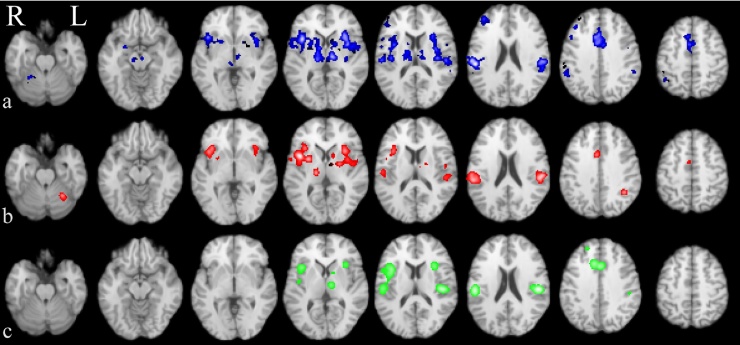
Coordinate based meta-analysis of neural response to cutaneous painful stimulation showing significant activation likelihood maps [pain activation] for healthy controls (a, blue), chronic pain cohorts (b, red) and hyperalgesia (c, green).

The CBMA of nociceptive brain activation in the HC group revealed all ⿿pain-matrix⿿ processing areas (thalamus, INS, cingulate, PFC, S1, S2, parahippocampal gyrus, brainstem nuclei, basal ganglia, Fig. 2a, Table S2.1). No single structure is reported by all studies. Right and left posterior insula (BA13) were the most consistent, each being reported in around 66% of studies. Other structures of the pain matrix were also among the most frequently reported. The bilateral BA40 (inferior parietal, S2) and left ACC (BA24) were reported in at least 1/3 studies. Ranked frequencies of the most often reported structures are given (blue in Fig. 4). Interestingly amygdala activation is reported in less than 5% of experiments.

The CBMA of CP studies revealed clusters of activation in 15 structures: bilateral thalamus, bilateral INS, left S2, left dorsal ACC (BA32), bilateral precentral (BA6&BA44) and postcentral gyri (BA40), right medial frontal gyrus (MFG), bilateral basal ganglia and left anterior cerebellum (Fig. 2b, Table S2.2).

The frequency plot (Fig. 4, red) depicts the most often reported activation sites in CP: the right posterior insula (BA13) was the most frequently reported (63% of all CP studies) followed by the left BA13 and the bilateral inferior parietal lobule (S2; BA40) in 37⿿50% of all studies. Bilateral putamen was reported by 34% of all CP studies.

**Fig. 3:**
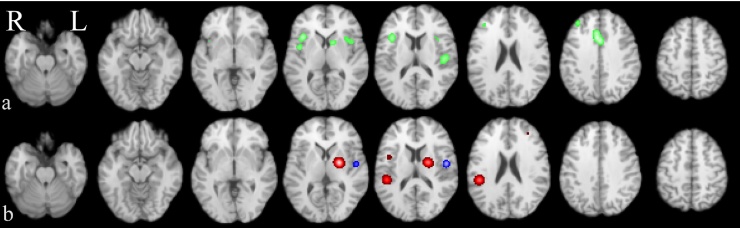
Coordinate based meta-analysis of significant aggregated contrasts between condition [pain activation during hyperalgesia > normalgesia] (MAC, a, green) and significant difference of activation likelihood maps between stimulation sites [most painful clinically affected (CS) > other site (OS)] (CMA b, red (CS > OS), blue (OS > CS)).

**Fig. 4:**
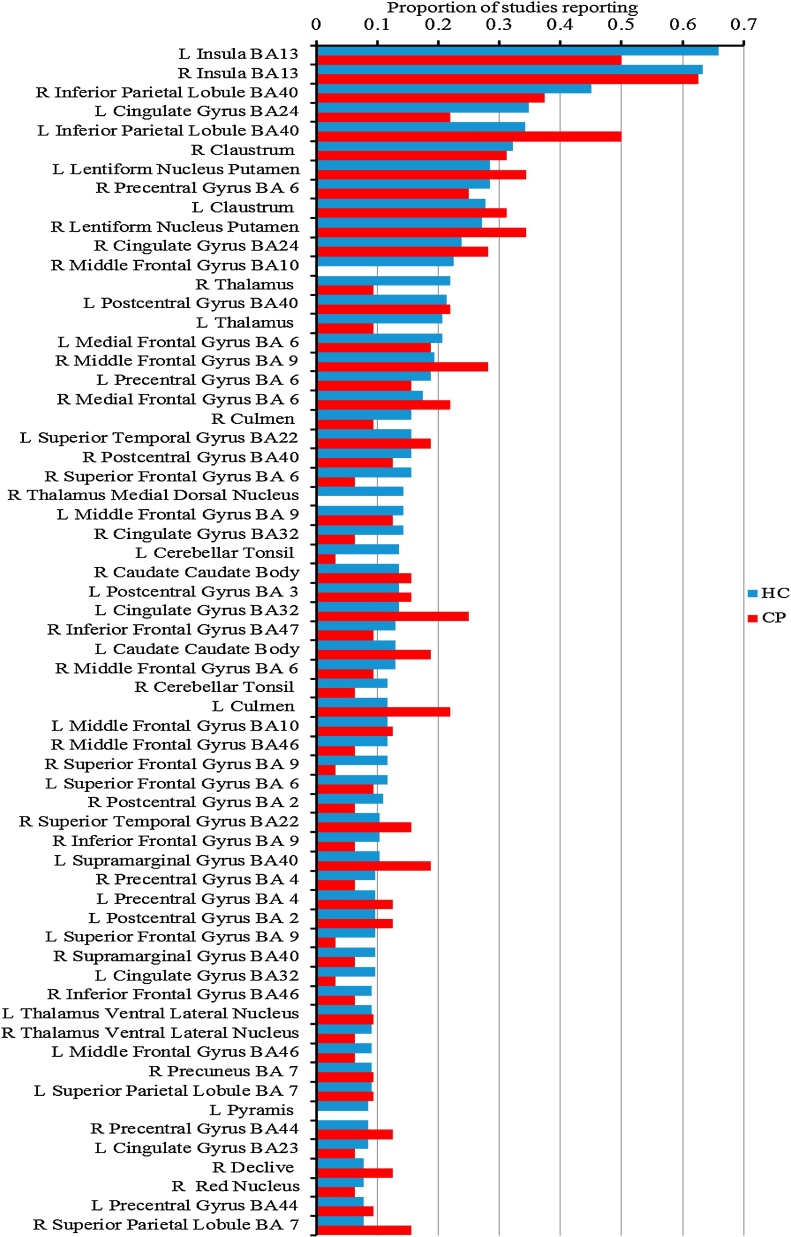
Frequency of reported structures in HC (blue) and CP (red) groups. Structures reported at least by 10% of the studies in HC group are displayed (For interpretation of the references to color in this figure legend, the reader is referred to the web version of this article.).

### 3.3 Does the neural pain response pattern differ between CP and HC?

CBMA in both HC and CP groups revealed similar clusters. No significant spatial differences in the activation pattern were found by CMA. However, there was a trend towards a significant difference just beyond the FCDR threshold (CP > HC, FCDR = 0.13) in the left supramarginal gyrus (BA40). A comparison using the omnibus test suggested subtle global differences reaching significance (*p* = 0.048).

We qualitatively compared the structures reported as activated in both groups (HC and CP) using a frequency plot (Fig. 4), and noticed that both groups reported activated structures with relatively similar frequencies.

While no statistically significant spatial differences in activation patterns were detected, this does not rule out the possibility that there are consistent spatial activation intensity differences. Therefore, MAC of reported comparisons of CP and HC (CP activation >HC activation, subgroup CP_HC_MAC_; Table 2) was performed, which did not reveal any significant clusters of activation intensity difference. In all but two of the included studies, the painful stimulation was applied to another part of the body that was remote from the site of clinical pain in the CP subjects. An additional MAC of hyperalgesia vs. normalgesia condition within CP subjects (HYPER_CP_MAC_) was also non-significant but only included three studies.

Due to the potential inhomogeneity introduced by the different pain stimulus modalities, we also compared HC and CP subgroups including only studies of mechanical pain stimulation (subgroups MECH_HC_ and MECH_CP_; Table 1); this resulted in the largest possible (28 vs. 22 studies respectively) modality specific subgroup analyses. No significant differences were found by either CMA below an FCDR of 0.1, and the omnibus test of difference was not significant (*p* = 0.79).

In line with our hypothesis of higher personal relevance of pain stimulation of the most painful clinically affected body part we compared the CS_CP_ and OS_CP_ subgroups. The CMA CS_CP_ vs. OS_CP_ revealed four clusters of more likely activation in the CS_CP_ group (left putamen; right posterior INS, right mid-INS and left middle frontal gyrus, Fig. 3b), and one cluster of more likely activation in the OS_CP_ group (precentral gyrus, Fig. 3b, Tables 2.3⿿2.5). The frequency plots of reported structures in the two CP sub-groups, and HC, are displayed in Fig. S1. Left putamen was reported by 50% of the CS_CP_ studies, 19% of the OS_CP_ studies and 28% of the HC studies.

#### 3.3.1 Is functional reorganisation in CP limited to neuropathic pain?

We analysed by CBMA the neural signature of pain in neuropathic pain patients (subgroup NEUR_CP_; Table 1), chronic conditions involving MSK pain (subgroup MSK_CP_; Table 1), and FM (subgroup FM_CP_; Table 1). The cerebral structures consistently activated in the three subgroups were not significantly different either by CMA or the omnibus test of difference (*p* > 0.05). Findings were also not significant when including FM in the neuropathic pain group.

#### 3.3.2 Can functional reorganisation in CP be modelled by hyperalgesia in HC?

The pain activation pattern in a model of peripheral and central sensitisation (experimental hyperalgesia in healthy controls, subgroup HYPER_HC_, Table 1) is displayed in green in Fig. 2c. It shows activations in left thalamus, bilateral INS, right dorsal ACC (BA32), right superior frontal gyrus and the left lentiform nucleus (Table S2.6). The frequency plots of reported structures activated by experimental hyperalgesia in HC are displayed in Fig. S2. Of note, left putamen was reported by 33% of HYPER_HC_ studies.

The CMA of hyperalgesia vs. normalgesia in HC did not elicit significant local or diffuse differences (omnibus test: *p* = 0.7).

#### 3.3.3 MAC of hyperalgesia vs. normalgesia in HC

Contrast data comparing experimental pain induced in the presence of hyperalgesia and experimental pain induced in normal condition (normalgesia) was available for extraction in eleven studies of which six used stimulus matched comparison (subgroup HYPER_MAC_, Table 2). MAC indeed revealed a multifocal effect of hyperalgesia on brain activation with significant clusters of increased activation in the dorsal ACC (BA32), bilateral INS (BA13), left insula (BA40), right IPL (S2, BA40), right middle frontal gyrus (BA9), and left striatum (Fig. 3a, Table S2.7). The activation pattern is highly similar to the pain signature revealed by CBMA of the CP and HC groups (Fig. 2a and b). These results suggest that hyperalgesia increases activation intensity in many of the known pain processing areas compared to normalgesia which may in part reflect the experimental design of stimulus intensity rather than perception matching in five studies. Nevertheless, when inspecting the paradigms of those studies contributing to significant clusters perception-based matching designs contributed to all clusters.

It should be noted however, that at least two of four studies (from the same centre) contributed to all of the significant clusters that possibly contain overlapping subject samples (Maihofner and Handwerker, 2005, Maihofner et al., 2004, Seifert et al., 2008, Seifert and Maihofner, 2007). As this could not be proved conclusively, no study was excluded.

#### 3.3.4 Can functional reorganisation in CP be modelled by low mood in HC?

MAC comparing experimental pain induced in the presence of low mood and experimental pain induced in the absence of low mood included data from only five studies (subgroup LM_HC_MAC_, Table 1). MAC did not detect regions of consistently increased or decreased activation in the presence of low mood. Activation foci for the low mood condition were only reported seperately in one study so no CBMA was possible.

## 4 Discussion

Coordinate based meta-analysis of 266 cutaneous pain fMRI studies demonstrated remarkably similar patterns with no significant spatial differences in nociceptive processing across all conditions of pain stimulation in chronic pain compared to healthy controls in normalgesia as well as after induced hyperalgesia. The subgroup of studies applying painful stimuli to the most painful clinically affected body part in CP, however, revealed that activation was significantly more likely in the left putamen, right mid and posterior insula and left middle frontal gyrus than if nociceptive stimuli are applied elsewhere. In contrast, there were no local differences in likelihood of activation between experimental hyperalgesia and normalgesia but activation intensity was upregulated in many of the pain processing areas including left anterior medial striatum and bilateral insula.

The cutaneous pain fMRI response pattern was very similar in healthy controls and chronic pain patients, and no spatial difference was detected by contrast meta-analysis; a finding reflected in the similarity of the per-structure activation report frequencies for the two groups. This finding was unexpected as functional studies are commonly referenced to evidence nociceptive neuroplasticity in chronic pain. The lack of a consistent shift of the spatial representation of nociceptive processing in clinical pain patients vs. controls is in stark contrast to previous coordinate based meta-analyses (Friebel et al., 2011, Lanz et al., 2011) that, however, contradict each other. Lanz et al. reported significantly more likely activated S2, contralateral SMA and ipsilateral cerebellum in patients vs. controls, but less likely activation in S1, insula, ACC, PFC, thalamus and cerebellum. By contrast, Friebel reported stronger convergence of activation in the left supramarginal gyrus, right anterior insula and left ACC in patients and reduced activation likelihood in the left posterior, right anterior insula, right SMA and S2. These discrepancies can be explained by methodological differences: Lanz et al. used a null hypothesis that coordinates were randomly distributed (Laird et al., 2005), while Friebel et al. used a more appropriate permutation of the grouping variable, but did not correct for multiple voxel-wise statistical tests. Another important limitation of previous pain CBMAs is lack of consideration for the correlation between coordinates reported by similar experiments using the same subjects; it is important that these coordinates are not treated as independent (Turkeltaub et al., 2012). Indeed, when rerunning the CBMA without appropriate merging, eight clusters were detected comparing the CP and HC groups (data not shown). Therefore, previously reported group differences might be explained by lack of rigorous data merging as required for any meta-analysis, or by inadequate statistical methodology.

Regardless of the appropriateness of statistical inferences made, CMA can only test for consistent differences in the pattern of reported activation between groups, and not for possible differences in activation intensity within shared activations. This can be addressed by aggregating reported activation foci detected by studies comparing groups, such as HC and CP. MAC of data from eighteen eligible studies did not reveal spatially consistent activation intensity differences between CP and healthy controls. Five pain fMRI studies comparing CP and HC failed to show significant results on direct comparison of the CP and HC groups. Furthermore, seven studies did not report the results of comparison between these groups. It is conceivable that this represents a publication bias as authors might have refrained from undertaking between group comparison due to expected low power or from reporting negative results, which either way would be in line with the negative MAC result.

Failure to observe a consistent localised differential activation pattern across the studied populations and experimental designs does not exclude possible subtle widespread differences in activation pattern, or differences limited to specific patient subgroups or experimental settings; for example a systematic anterior shift of insular activation was reported for neuropathic patients (Schweinhardt and Bushnell, 2010). Also, a recent detailed analysis demonstrated plasticity of the somatosensory system only in patients with chronic neuropathic pain (Gustin et al., 2012). Therefore, we additionally tested for global differences in the activation pattern between groups, and subgroups, using a more sensitive omnibus test (Tench et al., 2014). This suggested a subtle global differences across activation patterns between the HC and CP (*p* = 0.048). Importantly, functional reorganisation is likely moderated by pain etiology and phenotype, but the neuropathic and nociceptive subgroups were not detectably different from the HC group. Furthermore, there is some uncertainty how to classify patients with fibromyalgia (FM), and individual pain fMRI studies suggested hyperactivation of the insula which might reflect the postulated multisensory sensation syndrome in FM (Gracely et al., 2002). However, no differences between the subgroup of eight pain fMRI studies in FM and healthy controls were detected.

The experimental conditions are known to affect the pattern and intensity of fMRI response in healthy controls, so it is unclear how to best study brain plasticity in CP. Intuitively, eliciting clinical pain would be most relevant. However, as this is not always possible, we subgrouped CP studies according to whether the stimulus was applied on the clinically affected body part or remotely. When exposed to experimental painful stimulation of their most painful clinically affected body part, CP exhibited consistently higher likelihood of activation in the left putamen, left middle frontal gyrus, right posterior and mid INS. Post hoc analysis comparing activation patterns on clinical site stimulation to healthy controls revealed the two most significant stuctures were the left putamen and right INS, but these were not significant after correction for multiple comparisons. This is also suggested by the frequency plots showing putaminal activation in 28% of HC pain fMRI studies in contrast to 50% in CP exposed to nociception of the affected body part. We did not identify any other experimental factor (pain modality, cued vs. non cued, control condition) predicting the increased likelihood of putaminal activation.

The putamen is not considered part of the common neural signature of pain, with its main function being motor and implicit learning. Recently, however, a compelling case was made that the putamen may play a key role in co-ordinating nociceptive, sensory and cognitive-emotional pain processing (Starr et al., 2011). Such a role is foremost supported by the anatomical connectedness of the putamen; probabilistic tractography revealed that the putamen is not only interconnected with sensori-motor circuits but also nociceptive and attention areas including ACC, INS and thalamus, emotional and memory networks including the amygdala, hippocampus and substantia nigra (SN)/ventral tegmental area (VTA) (Starr et al., 2011). SN and VTA both receive direct afferent nociceptive information from the spinal cord via the parabrachial nucleus in the midbrain, and activate the putamen during pain (Bernard and Besson, 1990, Craig, 1995, Klop et al., 2005, Schneider, 1986, Vankova et al., 1992). Importantly, the putamen can shape activity in large areas of cortex via differentially modulating the levels of inhibition into the thalamus in both animals and humans (Alexander and Crutcher, 1990, Alexander et al., 1990, Chevalier and Deniau, 1990, Middleton and Strick, 2000, Mufson and Mesulam, 1984, Vogt et al., 1987). Mechanistic support also comes from a study in patients with putamenal lesions who demonstrated decreased pain sensitivity and widespread decreases in pain-related brain activation (Starr et al., 2011).

We propose that the putamen plays a specific role in the maladaptive state of chronic pain related to affective learning. Several functional neuroimaging studies showed putaminal/striatal activation in aversive learning (Delgado et al., 2008), disgust (Phillips et al., 1997), and hate (Zeki and Romaya, 2008). Putamen activation was also directly linked to learning of pain-related fear (Pohlack et al., 2012), with co-activation during early acquisition even in a rapid conditioned aversive learning paradigm using visceral pain as an unconditioned stimulus (Gramsch et al., 2014). A link of the observed increased putaminal activation in CP with aversive affective learning is also in line with recently reported coordinate based meta-analysis of fMRI studies on emotion regulation finding increased striatal activation during emotional upregulation, which was interpreted to be reflective of its role in affective learning and the initial stages of action preparation (Frank et al., 2014). Of note, most of the studies included in Frank et al. involve unpleasant stimuli, (Frank et al., 2014). The observed modified putaminal response to pain stimulation of clinically affected body parts might thus reflect implicit aversive learning and consecutive enhanced affective regulation (Baliki and Apkarian, 2015).

Intriguingly, putamen activation is an emerging hallmark of spontaneous pain processing in clinical pain as assessed by PET (Kulkarni et al., 2007) and arterial spin labelling (Howard et al., 2011, Kulkarni et al., 2007, Liu et al., 2013). These studies consistently reported increased blood flow and metabolism indexing neural activity in the left putamen in patients with on-going pain. Further supporting a key role for the putamen in pain augmentation comes from the observation of high incidence of pain in Parkinson⿿s characterised by putaminal dopaminergic denervation (Ford, 1998), putaminal dopaminergic deficit in chronic pain patients (Jaaskelainen et al., 2001), and an inverse link between D2 receptors and dopamine availability and pain sensitivity in healthy controls (Hagelberg et al., 2004). Taken together with our findings this provides evidence for a previously unrecognised key role of the putamen in maladaptive neuroplasticity in the chronic pain state, which is likely to reflect emotional upregulation. Our finding of modified putamen activity linked to pain stimulation of the affected body part highlights that functional reorganisation in CP is site-sepecific. This could possibly result from local and regional sensitisation as well as conditional augmentation which is dependent upon it⿿s contextual relevance in line with implicit aversive learning.

Noxious stimulation at the most painful clinically affected site in CP also induced more right posterior INS activation, which likely reflects CP induced hyperalgesia. In fact, the posterior insula is the most consistently reported brain activation site across all pain conditions and groups (frequency plots). This is in line with direct stimulation experiments showing that pain perception can only be elicited from the posterior insula (Ostrowsky et al., 2002). Our result concords with recent evidence for a close correlation of pain perception with regional blood flow increase in the posterior insula (Segerdahl et al., 2015). Posterior INS also encodes intensity of a pain stimulus (Frot et al., 1991) and is hence a plausible neural correlate of hyperalgesia. Peripheral and central sensitisation are increasingly recognised not only in neuropathic but also primary nociceptive chronic pain syndromes such as OA, leading to local and, to a lesser extent, remote hyperalgesia evidenced by reduced pain thresholds (Suokas et al., 2012). Given the core role of the posterior insula for pain perception, increased activation likely reflects hyperalgesia in chronic pain. Moreover, INS activation correlates with unpleasantesness of thermal hyperalgesia (Maihofner and Handwerker, 2005) and is involved in modulation of the affective aspect of sensory perception by pain expectation (Sawamoto et al., 2000). Multiple functional associations have been reported for the middle frontal gyrus (BA9), including attention to negative emotional stimuli (Kerestes et al., 2012). Interstingly, BA9 was also significantly more likely to be activated in clinical site vs. other site pain stimulation.

A main aim of our meta-analysis was to investigate the nature of maladaptive neuroplasticity in chronic pain using a mechanistically based approach. The most popular model of pain sensitisation is experimentally induced transient hyperalgesia in healthy volunteers using capsaicin to reduce pain thresholds locally and remotely for homo- and heteromodal nociception thus modelling both peripheral and central sensitisation (Woolf, 2011). Should the observed increased likelihood of putaminal, middle frontal and mid and posterior INS activation in CP stimulated at the clinical site be caused by sensitisation alone, we would expect a similar difference to emerge from experimental hyperalgesia. In contrast, we found no differences between the aggregate response pattern to cutaneous noxious stimuli in experimental hyperalgesic vs. normalgesic. The smallest *p* value for the left putamen from this CMA (HYPER_HC_ vs HC) was 0.014, which is far above the threshold for significance after correction for multiple comparisons. This is not surprising, as the frequency plots show left putamen and right INS being reported with close frequencies by studies in both groups: 33% HYPER_HC_ and 28% in the HC group (left putamen); 77% HYPER_HC_ and 66% in the HC (right INS). This is again in clear contradiction with previous CMA findings that described a higher activation likelihood of bilateral S2 and prefrontal cortex, right ACC, left basal ganglia and cerebellum in hyperalgesia/allodynia as well as reduced activation likelihood of the right insula, left ACC and prefrontal cortex, bilateral thalamus and bilateral basal ganglia (Lanz et al., 2011). As discussed above the fundamental differences in statistical inference explain the stark differences.

Intriguingly, while we found no spatial differences in the activation likelihood pattern using CMA, using MAC we found several clusters of consistently increased activation intensity as a neural correlate of central sensitisation. The observed hyperactivity pattern in experimental hyperalgesia includes many pain processing areas with the mid-left ACC, bilateral insula, right S2, left striatum in addition to the right middle frontal gyrus. The most parsimonous explanation would thus be that the brain correlate of central sensitisation is a generalised upregulation of pain processing. This in turn would be in line with the behavioral hyperalgesia and mechanistically with increased nociceptive signalling (Woolf, 2011). The pattern also closely resembles regions identified to encode perceived pain intensity (Favilla et al., 2014) including the salience network (anterior INS and ACC). Increased salience network activation furthermore accords well with the real world experience of heightened arousal and cognitive attention in hyperalgesia/allodynia induced by sunburn. The lack of altered activation likelihood, however, suggests that *transient* central sensitisation is insufficient to induce functional brain reorganisation. Moreover, the pattern of the neural pain response modulated by experimental cutaneous hyperalgesia is largely dissociated from that in CS_CP_ demonstrating that experimental hyperalgesia does not mimic neuroplasticity in clinical pain. Importantly, these dissociations cast doubt on the validity of experimental hyperalgesia as model for assessing analgesics for chronic pain conditions (Olesen et al., 2012).

When comparing the hyperalgesia pattern identified using MAC with the altered activation likelihood pattern in CS_CP_ only remote similarities can be noted. From 7 clusters in the hyperalgesia-normalgesia contrast, only two clusters showed proximity to the CS_CP_ pattern: the left lentiform nucleus cluster in hyperalgesia maps adjacent (more medial, anterior and inferiorly) to the left putamen maximum in CS_CP_; and secondly the right mid insula (BA13) in hyperalgesia locates anteriorly to the right mid insular cluster of increased activation likelihood in CS_CP_. Despite the predominant dissociation of experimental hyperalgesia and CS_CP_, the proximity of some activation foci point to possible partially shared mechanisms. Against the background of known pain sensitisation in both conditions, the different type of pain activation augmentation (increased activation intensity vs. likelihood) in adjacent regions might reflect the dynamics of transient to chronic central sensitisation. It is intriguing to speculate that regional hyperactivation as demonstrated by a model of central sensitisation over time may result in functional reorganisation indexed as increased activation likelihood in chronic pain.

The aggregate cutaneous pain response pattern identified from this coordinate based meta-analysis of 266 cutaneous pain fMRI studies is confirmatory of previous meta-analysis in healthy subjects reflecting the expected multidimensional neural networks contributing to the subjective pain experience. The consistently activated brain regions represent the so called neural signature of pain (Wager et al., 2013), formerly referred to as ⿿pain matrix⿿ (Melzack, 2001), and include the sensory nociceptive loop (brainstem, S1, S2, posterior insula, thalami), the salience network (ACC, anterior insula), prefrontal cortex and striatum. None of these brain activations are specific for pain (Iannetti and Mouraux, 2010), but the fine grained neural activation pattern identified through machine learning was shown to predict physical pain with 94% accuracy, and even analysis of activation patterns within single regions (dorsal ACC, anterior INS, S2 and post INS) achieved on average better than 75% sensitivity and specificity (Wager et al., 2013). This consistent neural signature of pain does, however, not translate into the same consistency of identified activation foci across pain fMRI studies included in our analysis. Our frequency plot analysis that is directly comparable to systematic reviews performed before advent of coordinate based spatial MA confirms that the posterior insula (right and left) is the most consistently reported structure. However, it is noteworthy that more than 1 in 3 fMRI pain studies do not report activation of the posterior insula. This highlights a remarkable sensitivity issue of current pain fMRI studies rather than a genuine variability of pain processing as functional imaging studies are known for very low statistical power (Button et al., 2013).

The main limitations of this coordinate based meta-analysis are the limited number, and heterogeneity, of chronic pain fMRI studies as well as inconsistent reporting. With 266 studies included this is one of the largest and arguably most rigorous pain fMRI CBMA to date in healthy volunteers but numbers in chronic pain patients and hyperalgesic conditions are lower. In particular, there were only few cutaneous pain fMRI studies in low mood, which reported contrasts only, precluding the possibility of mood-related CMA and preventing firm interpretation of the negative results of the low vs. normal mood meta-analysis of contrasts. Importantly, there is substantial variation across studies in which criteria were applied to report ⿿peak activation⿿ depending on the analysis methods used, in particular the thresholds used which varied between *P* < 0.001 uncorrected, FDR and FWE correction. This reflects the lack of consensus on standards, use of different software packages and versions and incomplete reporting in the field of functional neuroimaging (reviewed by Carp (2012)). Also, only one in five of the included studies reported coordinates for functional deactivation precluding a meta-analysis of this important feature of nociceptive response. This and the lack of included CBF studies of spontaneous pain prevent a direct comparison with the meta-analysis reported by (Peyron et al., 2000), which was largely based on PET studies. We chose to limit our meta-analysis to fMRI as strong method dependent differences were reported previously (Peyron et al., 2000).

Another important limitation is the incomplete reporting of pain intensity which results in the inability to control for perceived pain intensity between subgroups. Nevertheless, in those studies where the group comparisons were directly reported and extracted (for the MAC analyses) we found a balanced count of studies using percept or stimulus intensity matched paradigms. As these numbers were low, additional subgroup analyses were not carried out, Thus we cannot disentangle the effects of transient central sensitisation from experimentally designed differences in pain intensity encoding. We did not extract pain anticipatory brain activity, which might reveal additional CP specific alterations as there were so few studies reporting on this in CP cohorts.

We limited this coordinate based meta-analysis to the more commonly used cutaneous pain stimuli, and can thus not generalise our findings to deep pain stimulation of muscle or viscera. Hence we also did not include muscle or visceral experimental hyperalgesia, which conceivably might heighten pain unpleasantness and thus might more closely match the neural signature of chronic pain.

In general, we found that only a minority of CP studies and not all experimental hyperalgesia studies reported results (co-ordinates) from between condition/group comparisons. This is highly recommended for future pain fMRI studies as MAC is a powerful alternative to CMA for several reasons: (i) the direct between group or condition comparison is an effective way to controlling for unwanted heterogeneity resulting from technical and experimental specificities between studies, (ii) the advantages of careful matching of case control studies are only preserved in MAC but not CMA, and (iii) MAC is sensitive to differences in activation strength. Current drives for publicly available neuroimaging repositories will have the additional advantage for large pooled image based analysis with the potential of further advances over CBMA (Salimi-Khorshidi et al., 2009) provided effective and validated means for controlling study-and scanner specific bias become available.

## 5 Conclusion

Coordinate based meta-analysis of superficial pain fMRI studies using statistically rigorous methodology revealed remarkably similar activation patterns in healthy controls during normalgesia and hyperalgesia and chronic pain patients. However, we describe a consistent pattern of hyperactivation in experimental hyperalgesia as model of transient central sensitisation. Also, we identified increased likelihood of left putaminal, right middle frontal gyrus and mid/posterior insular activation when stimulating the clinically affected site in CP, which we interpret to reflect neuroplasticity linked to chronic sensitisation augmented by aversive contextual learning.

## Appendix A Supplementary data

Supplementary File

Supplementary Fig. S1 Most frequently reported structures in the CP sub-groups (red-CSCP, yellow-OSCP). HC (blue) are added for comparison. Structures reported at least by 10% of the studies in one group are displayed.

Supplementary Fig. S2 Frequency of reported structures in Hyperalgesia (green) and HC (blue) groups. Structures reported at least by 10% of the studies in one group are displayed.
