# Supplementary material for: Functional reorganisation in chronic pain and neural correlates of pain sensitisation: A coordinate based meta-analysis of 266 cutaneous pain fMRI studies
Source: Neurosci Biobehav Rev. 2016 Sep;68:120–33. doi: 10.1016/j.neubiorev.2016.04.001 (PMC5554296; doi:10.1016/j.neubiorev.2016.04.001)
Supplement: Supplementary Table S1 [file mmc1.docx]

| Table S1 - Papers included in the coordinate based meta-analysis  **Patient abbreviations:** PHN – Postherpetic neuralgia, CRPS – Complex Regional Pain Syndrome, FM – Fibromyalgia, SM – Syringomyelia, TNP – Trigeminal Neuralgia, CBP – Chronic Back Pain, BMD – Burning Mouth Disorder, NP – Neuropathic pain, OA – Osteoarthritis, MOH – Medication Overuse Headache, M – Migraine, DN – Diabetic Neuropathy, VVS – Vulvar Vestibulitis Syndrome. **Subject groups’ abbreviations** are listed in the Results section in the main text. References are listed in the Supplementary Reference File | | | | | | | | | | | |
| --- | --- | --- | --- | --- | --- | --- | --- | --- | --- | --- | --- |
| **Author** | **Subject group / modality** | **Healthy volunteer or patient** | ***N*** | **Field strength** | **Pain stimulus** | **Side** | **Contrast** | **Cued?** | **Standard space (MNI/Tal)** | **Table(s) used** | **Number of foci** |
| ([Albanese et al., 2007](#_ENREF_1)) | HC, THER_HC_, RIGHT_HC_, INNOC_HC_, CUED_HC_ | Healthy Volunteer | 8 | 1.5T | Heat | Right | Vs. innocuous | Cued | MNI | Table 2 and 3 | 12 |
| ([Albuquerque et al., 2006](#_ENREF_2)) | HC, THER_HC_, RIGHT_HC_, INNOC_HC_, NCUED_HC_ | Healthy Volunteer | 8 | 1.5T | Heat | Right | Vs. innocuous | No cue | Tal | Table 7 | 10 |
|  | CP, NEUR_CP_, CS_CP_, NOX_CP_, CP_HC_MAC_ | Patient - BMD | 8 | 1.5T | Heat | Right | Vs. innocuous, Patient vs. Healthy volunteer | No cue | Tal | Table 5, 6 | 6, 4 |
| ([Apkarian et al., 2000](#_ENREF_3)) | HC, THER_HC_, RIGHT_HC_, INNOC_HC_, NCUED_HC_ | Healthy Volunteer | 7 | 1.5T | Heat | Right | Vs. innocuous | No cue | Tal | Table 1 | 3 |
| ([Atlas et al., 2010](#_ENREF_4)) | HC, THER_HC_, LEFT_HC_, INNOC_HC_, CUED_HC_ | Healthy Volunteer | 18 | 1.5T | Heat | Left | Vs. innocuous | Cue | MNI | Table S1. | 7 |
| ([Baliki et al., 2006](#_ENREF_5)) | HC, THER_HC_, INNOC_HC_, NCUED_HC_ | Healthy Volunteer | 11 | 3.0T | Heat | Central | Vs. innocuous | No cue | MNI | Table S4 | 16 |
|  | CP, MSK_CP_, CS_CP_, NOX_CP_ | Patient- CBP | 11 | 3.0T | Heat | Central | Vs. innocuous | No cue | MNI | Table S4 | 10 |
| ([Baliki et al., 2008](#_ENREF_7)) | CP, MSK_CP_, CS_CP_, NOX_CP_, MECH_CP_ | Patient – OA | 5 | 3.0T | Pressure | Mixed | Vs. rest | No cue | MNI | Table 4 | 13 |
| ([Baliki et al., 2010](#_ENREF_6)) | HC, THER_HC_, INNOC_HC_, NCUED_HC_ | Healthy Volunteer | 16 | 3.0T | Heat | Central | Vs. innocuous | No cue | MNI | Table S4 | 17 |
|  | CP, MSK_CP_, CS_CP_, NOX_CP_ | Patient – CBP | 16 | 3.0T | Heat | Central | Vs. innocuous | No cue | MNI | Table S4 | 17 |
| ([Becerra et al., 1999](#_ENREF_10)) | HC, THER_HC_, LEFT_HC_, INNOC_HC_, NCUED_HC_ | Healthy Volunteer | 6 | 1.5T | Heat | Left | Vs. innocuous | No cue | Tal | Table 1 | 14 |
| ([Becerra et al., 2001](#_ENREF_8)) | HC, THER_HC_, LEFT_HC_, INNOC_HC_, NCUED_HC_ | Healthy Volunteer | 8 | 1.5T | Heat | Left | Vs. innocuous | No cue | Tal | Table 1 and 2 | 81 |
| ([Becerra et al., 2006](#_ENREF_9)) | CP, NEUR_CP_, CS_CP_, ALDN_CP_, NOX_CP_, MECH_CP_, CP_HC_MAC_ | Patient – TNP | 6 | 3.0T | Heat, Cold and Tactile | Right | Vs. innocuous, Patient vs. Healthy Volunteer, Hyperalgesia vs. normalgesia | No cue | MNI | Table 2, 3 and 4. Supplemental Table 1A, 2A and 3A | 12, 14, 6, 20, 22 and 20. |
| ([Berna et al., 2010](#_ENREF_11)) | LM_HC_ | Healthy Volunteer | 20 | 3.0T | Heat | Left | Vs. rest | Cue | MNI | Table S3 | 15 |
| ([Bingel et al., 2002](#_ENREF_13)) | HC, THER_HC_, REST_HC_, NCUED_HC_ | Healthy Volunteer | 14 | 1.5T | Heat | Mixed | Vs. rest | No cue | MNI | Table 1 | 12 |
| ([Bingel et al., 2003](#_ENREF_14)) | HC, THER_HC_, RIGHT_HC_, LEFT_HC_, REST_HC_, NCUED_HC_ | Healthy Volunteer | 14 | 1.5T | Heat | Mixed | Vs. rest | No cue | MNI | Table 1 | 20 |
| ([Bingel et al., 2006](#_ENREF_12)) | HC, THER_HC_, RIGHT_HC_, CUED_HC_ | Healthy Volunteer | 19 | 1.5T | Heat | Mixed | Vs. rest | Cue | MNI | Table 1 | 17 |
| ([Bingel et al., 2007a](#_ENREF_15)) | HC, ELEC_HC_, REST_HC_, NCUED_HC_ | Healthy Volunteer | 16 | 3.0T | Electrical | Mixed | Vs. rest | No cue | MNI | Table 1 | 11 |
| ([Bingel et al., 2007b](#_ENREF_16)) | HC, THER_HC_, LEFT_HC_, INNOC_HC_, NCUED_HC_ | Healthy Volunteer | 20 | 3.0T | Heat | Left | Vs. innocuous | No cue | MNI | Table 1 | 21 |
| ([Bingel et al., 2011](#_ENREF_17)) | HC, THER_HC_, RIGHT_HC_, REST_HC_, CUED_HC_ | Healthy Volunteer | 22 | 3.0T | Heat | Right | Vs. rest | Cue | MNI | Table S1 | 20 |
| ([Bogdanov et al., 2015](#_ENREF_18)) | HC, THERHC, LEFTHC, NCUEDHC | Healthy Volunteer | 24 | 3.0T | Heat | Left | - | No cue | MNI | Table 1 | 53 |
| ([Boly et al., 2007](#_ENREF_19)) | HC, THER_HC_, LEFT_HC_, NCUED_HC_ | Healthy Volunteer | 24 | 3.0T | Heat | Left | - | No cue | MNI | Table 1 and 2 | 7 and 10 |
| ([Bornhovd et al., 2002](#_ENREF_20)) | HC, THER_HC_, LEFT_HC_, NCUED_HC_ | Healthy Volunteer | 9 | 1.5T | Heat | Left | - | No cue | MNI | Table 1 | 18 |
| ([Botvinick et al., 2005](#_ENREF_21)) | HC, THER_HC_, LEFT_HC_, INNOC_HC_, CUED_HC_ | Healthy Volunteer | 12 | 1.5T | Heat | Left | Vs. innocuous | Cue | MNI | Table 1 | 11 |
| ([Brinkmeyer et al., 2010](#_ENREF_22)) | HC, THER_HC_, LEFT_HC_, REST_HC_, NCUED_HC_ | Healthy Volunteer | 10 | 3.0T | Heat | Left | Vs. rest | No cue | MNI | Table 1 | 15 |
| ([Brooks et al., 2002](#_ENREF_25)) | HC, THER_HC_, RIGHT_HC_, LEFT_HC_, INNOC_HC_, NCUED_HC_ | Healthy Volunteer | 18 | 1.5T | Heat | Mixed | Vs. innocuous | No cue | Tal | Table 1 | 23 |
| ([Brooks et al., 2003](#_ENREF_24)) | HC, THER_HC_, RIGHT_HC_, INNOC_HC_, HYPER_HC_ | Healthy Volunteer | 16 | 1.5T | Heat | Right | Vs. innocuous | - | Tal | Table 1 | 11 |
| ([Brooks et al., 2005](#_ENREF_23)) | HC, THER_HC_, RIGHT_HC_, REST_HC_, NCUED_HC_ | Healthy Volunteer | 14 | 3.0T | Heat | Right | Vs. rest | No cue | MNI | Table 1 | 36 |
| ([Buchel et al., 2002](#_ENREF_26)) | HC, THER_HC_, LEFT_HC_, REST_HC_, NCUED_HC_ | Healthy Volunteer | 9 | 1.5T | Heat | Left | Vs. rest | No cue | MNI | Table 1 | 8 |
| ([Carlsson et al., 2006](#_ENREF_27)) | HC, ELEC_HC_, RIGHT_HC_, INNOC_HC_, CUED_HC_ | Healthy Volunteer | 9 | 1.5T | Electrical | Right | Vs. innocuous | Cue | Tal | Table 1 | 24 |
| ([Christmann et al., 2007](#_ENREF_28)) | HC, ELEC_HC_, RIGHT_HC_, REST_HC_, NCUED_HC_ | Healthy Volunteer | 6 | 1.5T | Electrical | Right | Vs. rest | No cue | Tal | Table 2 | 29 |
| ([Coghill et al., 2003](#_ENREF_29)) | HC, THER_HC_, RIGHT_HC_, INNOC_HC_, NCUED_HC_ | Healthy Volunteer | 17 | 1.5T | Heat | Right | Vs. innocuous | No cue | Tal | Table 1 | 7 |
| ([Cole et al., 2006](#_ENREF_30)) | HC, MECH_HC_, RIGHT_HC_, INNOC_HC_, NCUED_HC_ | Healthy Volunteer | 15 | 1.5T | Pressure | Right | Vs. innocuous | No cue | MNI | Supplementary Table 1 | 21 |
| ([Cole et al., 2010](#_ENREF_31)) | HC, MECH_HC_, RIGHT_HC_, INNOC_HC_, NCUED_HC_ | Healthy Volunteer | 30 | 1.5T | Pressure | Right | Vs. innocuous | No cue | MNI | Table 2 | 51 |
| ([Cook et al., 2004](#_ENREF_32)) | HC, THER_HC_, LEFT_HC_, INNOC_HC_, NCUED_HC_ | Healthy Volunteer | 9 | 1.5T | Heat | Left | Vs. innocuous | No cue | Tal | Table 2 | 6 |
|  | CP, FM_CP_, RS_CP_ | Patient – FM | 9 | 1.5T | Heat | Left | Vs. innocuous | No cue | Tal | Table 3 | 5 |
| ([Corradi-Dell'Acqua et al., 2011](#_ENREF_33)) | HC, THER_HC_, RIGHT_HC_, INNOC_HC_, CUED_HC_ | Healthy Volunteer | 28 | 3.0T | Heat | Right | Vs. innocuous | Cue | MNI | Table 2 | 26 |
| ([Davis et al., 2002](#_ENREF_34)) | HC, THER_HC_, RIGHT_HC_, REST_HC_, NCUED_HC_ | Healthy Volunteer | 7 | 1.5T | Cold | Right | Vs. rest | No cue | Tal | Table 1 | 19 |
| ([de la Fuente-Sandoval et al., 2010](#_ENREF_36)) | HC, THER_HC_, LEFT_HC_, INNOC_HC_, NCUED_HC_ | Healthy Volunteer | 13 | 3.0T | Heat | Left | Vs. innocuous | No cue | MNI | Table 2 | 8 |
| ([de la Fuente-Sandoval et al., 2012](#_ENREF_35)) | HC, THER_HC_, LEFT_HC_, INNOC_HC_, NCUED_HC_ | Healthy Volunteer | 13 | 3.0T | Heat | Left | Vs. innocuous | No cue | MNI | Table 2 | 8 |
| ([de Leeuw et al., 2006](#_ENREF_37)) | HC, THER_HC_, LEFT_HC_, INNOC_HC_, NCUED_HC_ | Healthy Volunteer | 9 | 1.5T | Heat | Left | Vs. innocuous | No cue | Tal | Table 1 | 16 |
| ([Derbyshire et al., 2004](#_ENREF_39)) | HC, THER_HC_, RIGHT_HC_, REST_HC_, NCUED_HC_ | Healthy Volunteer | 8 | 3.0T | Heat | Right | Vs. rest | No cue | MNI | Table 1 | 14 |
| ([Derbyshire and Osborn, 2009](#_ENREF_38)) | HC, THER_HC_, INNOC_HC_, NCUED_HC_ | Healthy Volunteer | 12 | 3.0T | Heat | Mixed | Vs. innocuous | No cue | MNI | Table 1 | 8 |
| ([Diers et al., 2012](#_ENREF_40)) | CP, FM_CP_, RS_CP_, NOX_CP_, MECH_CP_ | Patient – FM | 10 | 1.5T | Pressure | Left | Vs. rest | No cue | MNI | Table 2 | 5 |
| ([Downar et al., 2003](#_ENREF_41)) | HC, ELEC_HC_, RIGHT_HC_, REST_HC_, NCUED_HC_ | Healthy Volunteer | 10 | 1.5T | Electrical | Right | Vs. rest | No cue | Tal | Table 1 | 19 |
| ([Dube et al., 2009](#_ENREF_42)) | HC, THER_HC_, LEFT_HC_, INNOC_HC_, NCUED_HC_ | Healthy Volunteer | 12 | 1.5T | Heat | Left | Vs. innocuous | No cue | MNI | Table 1 and 2 | 41 |
| ([Ducreux et al., 2006](#_ENREF_43)) | HC, THER_HC_, RIGHT_HC_, REST_HC_, NCUED_HC_ | Healthy Volunteer | 6 | 1.5T | Cold | Right | Vs. rest | No cue | MNI | Table 4 | 25 |
|  | CP, NEUR_CP_, CS_CP_, ALDN_CP_, MECH_CP_ | Patient – SM | 6 | 1.5T | Cold and Pressure | Right | Vs. rest | No cue | MNI | Table 4 and 5 | 35 |
| ([Dunckley et al., 2005](#_ENREF_44)) | HC, THER_HC_, LEFT_HC_, REST_HC_, NCUED_HC_ | Healthy Volunteer | 10 | 3.0T | Heat | Left | Vs. rest | No cue | MNI | Table 1 | 37 |
| ([Fairhurst et al., 2007](#_ENREF_45)) | HC, THER_HC_, LEFT_HC_, REST_HC_, CUED_HC_ | Healthy Volunteer | 12 | 3.0T | Heat | Left | Vs. rest | Cue | MNI | Table 2 | 16 |
| ([Ferraro et al., 2012](#_ENREF_46)) | HC, MECH_HC_, LEFT_HC_, REST_HC_, NCUED_HC_ | Healthy Volunteer | 9 | 1.5T | Pressure | Left | Vs. rest | No cue | MNI | Table 2 | 8 |
|  | CP, CS_CP_, NOX_CP_, MECH_CP_ | Patient-MOH | 9 | 1.5T | Pressure | Left | Vs. rest | No cue | MNI | Table 2 | 17 |
| ([Ferretti et al., 2003](#_ENREF_47)) | HC, ELEC_HC_, RIGHT_HC_, REST_HC_, NCUED_HC_ | Healthy Volunteer | 8 | 1.5T | Electrical | Right | Vs. rest | No cue | Tal | Table 1 | 7 |
| ([Forkmann et al., 2013](#_ENREF_48)) | HC, THER_HC_, LEFT_HC_, INNOC_HC_, CUED_HC_ | Healthy Volunteer | 24 | 3.0T | Heat | Left | Vs. innocuous | Cue | MNI | Table 1 | 14 |
| ([Frankenstein et al., 2001](#_ENREF_49)) | HC, THER_HC_, RIGHT_HC_, REST_HC_, CUED_HC_ | Healthy Volunteer | 10 | 1.5T | Cold | Right | Vs. rest | Cue | MNI | Table 1 | 10 |
| ([Freund et al., 2007](#_ENREF_51)) | HC, ELEC_HC_, RIGHT_HC_, LEFT_HC_, REST_HC_, NCUED_HC_ | Healthy Volunteer | 15 | 1.5T | Electrical | Mixed | Vs. rest | No cue | MNI | Table 2 | 11 |
| ([Freund et al., 2009](#_ENREF_50)) | HC, THER_HC_, RIGHT_HC_, LEFT_HC_, REST_HC_, NCUED_HC_ | Healthy Volunteer | 15 | 1.5T | Heat | Mixed | Vs. rest | No cue | MNI | Table 2 | 10 |
| ([Freund et al., 2011](#_ENREF_52)) | CP, NEUR_CP_, RS_CP_, NOX_CP_ | Patient – CRPS | 10 | 1.5T | Electrical | Mixed | Vs. rest | No cue | MNI | Table 2 | 18 |
| ([Gard et al., 2012](#_ENREF_53)) | HC, ELEC_HC_, LEFT_HC_, REST_HC_, CUED_HC_ | Healthy Volunteer | 34 | 1.5T | Electrical | Left | Vs. rest | Cue | Tal | Table 1 and 2 | 27 |
| ([Geha et al., 2008](#_ENREF_54)) | CP, NEUR_CP_, CS_CP_, ALDN_CP_, MECH_CP_ | Patient – PHN | 11 | 3.0T | Pressure | Mixed | Vs. rest | No cue | MNI | Table 1 | 14 |
| ([Gelnar et al., 1999](#_ENREF_55)) | HC, THER_HC_, RIGHT_HC_, INNOC_HC_, CUED_HC_ | Healthy Volunteer | 9 | 1.5T | Heat | Right | Vs. innocuous | Cue | Tal | Table 3 | 9 |
| ([Geuze et al., 2007](#_ENREF_56)) | HC, THER_HC_, RIGHT_HC_, INNOC_HC_, NCUED_HC_ | Healthy Volunteer | 12 | 1.5T | Heat | Right | Vs. innocuous | No cue | Tal | Table 2 | 15 |
| ([Giesecke et al., 2006](#_ENREF_57)) | CP_HC_MAC_ | Patient-CBP and healthy volunteers | 22 | 1.5T | Pressure | Left | Patient vs. Healthy volunteer | No cue | Tal | Table 2 | 4 |
| ([Godinho et al., 2012](#_ENREF_58)) | HC, ELEC_HC_, INNOC_HC_, CUED_HC_ | Healthy Volunteer | 16 | 1.5T | Electrical | Mixed | Vs. innocuous | Cue | Tal | Table 1 | 16 |
| ([Gopinath et al., 2012](#_ENREF_59)) | HC, THER_HC_, RIGHT_HC_, INNOC_HC_, NCUED_HC_ | Healthy Volunteer | 14 | 3.0T | Heat | Right | Vs. innocuous | No cue | Tal | Table 2 | 33 |
| ([Gracely et al., 2002](#_ENREF_60)) | CP_HC_MAC_ | Patient-FM, Healthy Volunteer | 16, 16 | 1.5T | Pressure | Left | Vs. rest, Patient vs. Healthy Volunteer | No cue | Tal | Table 3 | 13 |
| ([Grant et al., 2011](#_ENREF_61)) | HC, THER_HC_, LEFT_HC_, INNOC_HC_, CUED_HC_ | Healthy Volunteer | 13 | 3.0T | Heat | Left | Vs. innocuous | Cue | Tal | Table S1 | 10 |
| ([Gundel et al., 2008](#_ENREF_62)) | HC, THER_HC_, LEFT_HC_, INNOC_HC_, NCUED_HC_ | Healthy Volunteer | 12 | 1.5T | Heat | Left | Vs. innocuous | No cue | MNI | Table 2 | 13 |
| ([Herde et al., 2007](#_ENREF_63)) | HC, THER_HC_, RIGHT_HC_, INNOC_HC_, NCUED_HC_ | Healthy Volunteer | 8 | 1.5T | Heat | Right | Vs. innocuous | No cue | Tal | Table 2 | 28 |
| ([Hiramatsu et al., 2014](#_ENREF_64)) | CP_HC_MAC_ | Patient-OA | 12 | 3.0T | Electrical | Right | - | No cue | MNI | Table 2 | 7 |
| ([Hohmeister et al., 2010](#_ENREF_65)) | HC, THER_HC_, LEFT_HC_, REST_HC_, NCUED_HC_ | Healthy Volunteer | 9 | 1.5T | Heat | Left | Vs. rest | No cue | MNI | Table 3 | 13 |
| ([Iannilli et al., 2008](#_ENREF_66)) | HC, ELEC_HC_, RIGHT_HC_, REST_HC_, NCUED_HC_ | Healthy Volunteer | 18 | 1.5T | Electrical | Right | Vs. rest | No cue | Tal | Table 2 | 24 |
| ([Ibinson and Vogt, 2013](#_ENREF_67)) | HC, ELECHC, RIGHTHC, NCUEDHC | Healthy Volunteer | 15 | 3.0T | Electrical | Right | - | No cue | MNI | Tables 1 & 3 | 19 |
| ([Jantsch et al., 2005](#_ENREF_68)) | HC, ELEC_HC_, LEFT_HC_, REST_HC_, NCUED_HC_ | Healthy Volunteer | 8 | 1.5T | Electrical | Left | Vs. rest | No cue | Tal | Table 2 | 18 |
| ([Jensen et al., 2010](#_ENREF_70)) | CP, FM_CP_, RS_CP_, NOX_CP_, MECH_CP_ | Patient-FM | 83 | 1.5T | Pressure | Left | Vs. innocuous | No cue | MNI | Table 2 | 10 |
| ([Jensen et al., 2012](#_ENREF_69)) | CP, FM_CP_, RS_CP_, NOX_CP_, MECH_CP_ | Patient-FM | 40 | 1.5T | Pressure | Right | Vs. innocuous | No cue | MNI | Table 2 | 6 |
| ([Kamping et al., 2013](#_ENREF_72)) | HC, THER_HC_, LEFT_HC_, NCUED_HC_ | Healthy Volunteer | 16 | 3.0T | Heat | Left | - | No cue | MNI | Table 2 | 15 |
|  | CP, FM_CP_, RS_CP_, NOX_CP_, | Patient-FM | 16 | 3.0T | Heat | Left | - | No cue | MNI | Table 2 | 2 |
| ([Kakeda et al., 2010](#_ENREF_71)) | HC, THER_HC_, REST_HC_, NCUED_HC_ | Healthy Volunteer | 12 | 3.0T | Cold | Mixed | Vs. rest | No cue | MNI | Table 2 | 11 |
| ([Kim et al., 2011](#_ENREF_73)) | HC, MECH_HC_, LEFT_HC_, REST_HC_, NCUED_HC_ | Healthy Volunteer | 22 | 3.0T | Pressure | Left | Vs. rest | No cue | MNI | Supplementary Table 1 | 18 |
|  | CP, FM_CP_, RS_CP_, NOX_CP_, MECH_CP_ | Patient-FM | 19 | 3.0T | Pressure | Left | Vs. rest | No cue | MNI | Supplementary Table 1 | 14 |
| ([Kim et al., 2013](#_ENREF_74)) | HC, MECH_HC_, LEFT_HC_, NCUED_HC_ | Healthy Volunteer | 11 | 3.0T | Pressure | Left | - | No cue | MNI | Table 1 | 9 |
|  | CP, FM_CP_, RS_CP_, NOX_CP_, MECH_CP_, CP_HC_MAC_ | Patient-FM | 21 | 3.0T | Pressure | Left | - | No cue | MNI | Table 2 and 3 | 13, 8 |
| ([Kobayashi et al., 2009](#_ENREF_75)) | HC, MECH_HC_, RESTt_HC_, NCUED_HC_ | Healthy Volunteer | 8 | 3.0T | Pressure | Central | Vs. rest | No cue | Tal | Table 3 | 4 |
|  | CP, MSK_CP_, CS_CP_, NOX_CP_, MECH_CP_ | Patient-CBP | 8 | 3.0T | Pressure | Central | Vs. rest | No cue | Tal | Table 3 | 8 |
| ([Kong et al., 2006](#_ENREF_78)) | HC, THER_HC_, RIGHT_HC_, NCUED_HC_ | Healthy Volunteer | 16 | 3.0T | Heat | Right | High vs. low pain | No cue | MNI | Table 1 | 13 |
| ([Kong et al., 2010](#_ENREF_77)) | HC, THER_HC_, RIGHT_HC_, INNOC_HC_, CUED_HC_ | Healthy Volunteer | 61 | 3.0T | Heat | Right | Vs. innocuous | Cue | MNI | Table 2 | 12 |
| ([Kong et al., 2013](#_ENREF_76)) | HC, THER_HC_, RIGHT_HC_, REST_HC_, CUED_HC_ | Healthy Volunteer | 46 | 3.0T | Heat | Right | Vs. rest | Cue | MNI | Table 1 | 15 |
| ([Koyama et al., 2005](#_ENREF_79)) | HC, THER_HC_, RIGHT_HC_, INNOC_HC_, NCUED_HC_ | Healthy Volunteer | 10 | 1.5T | Heat | Right | Vs. innocuous | No cue | Tal | Supplementary Table 1 | 17 |
| ([Kucyi et al., 2013](#_ENREF_80)) | HC, ELEC_HC_, LEFT_HC_, NCUED_HC_ | Healthy Volunteer | 51 | 3.0T | Electrical | Left | - | No cue | MNI | Supplementary Table 2 | 22 |
| ([Kurata et al., 2002](#_ENREF_82)) | HC, THER_HC_, RIGHT_HC_, INNOC_HC_, NCUED_HC_ | Healthy Volunteer | 5 | 3.0T | Heat | Right | Vs. innocuous | No cue | Tal | Table 2 | 18 |
| ([Kurata et al., 2005](#_ENREF_81)) | HC, THER_HC_, RIGHT_HC_, INNOC_HC_, NCUED_HC_ | Healthy Volunteer | 6 | 3.0T | Heat | Right | Vs. innocuous | No cue | Tal | Table 2 | 2 |
| ([La Cesa et al., 2014](#_ENREF_83)) | HC, THER_HC_, LEFT_HC_, NCUED_HC_ | Healthy Volunteer | 12 | 3.0T | Cold | Left | - | No cue | MNI | Table 1 | 24 |
| ([Landgrebe et al., 2008](#_ENREF_84)) | HC, THER_HC_, LEFT_HC_, REST_HC_, CUED_HC_ | Healthy Volunteer | 15 | 3.0T | Heat | Left | Vs. rest | Cue | MNI | Table 2 | 10 |
| ([Lebel et al., 2008](#_ENREF_85)) | CP, NEUR_CP_, CS_CP_, ALDN_CP_, MECH_CP_ | Patient-CRPS | 8 | 3.0T | Pressure | Mixed | Vs. innocuous | No cue | MNI | Table 5 | 13 |
| ([Lee et al., 2008](#_ENREF_86)) | HYPER_MAC_ | Healthy Volunteer | 15 | 3.0T | Pressure | Right | Mechanical hyperalgesia vs. mechanical normalgesia | No cue | MNI | Supplemental Table 1 | 17 |
| ([Liljencrantz et al., 2013](#_ENREF_87)) | HYPER_MAC_ | Healthy Volunteer | 18 | 3.0T | Pressure | Left | Mechanical hyperalgesia vs. mechanical normalgesia | Cue | MNI | Table 1 | 17 |
| ([Lloyd et al., 2008](#_ENREF_88)) | HC, MECH_HC_, REST_HC_, NCUED_HC_ | Healthy Volunteer | 17 | 1.5T | Pressure | Central | Vs. rest | No cue | MNI | Table 1 | 9 |
|  | CP, MSK_CP_, CS_CP_, ALDN_CP_, MECH_CP_ | Patient-CBP | 28 | 1.5T | Pressure | Central | Vs. rest | No cue | MNI | Table 1 | 21 |
| ([Lloyd et al., 2014](#_ENREF_89)) | CP, MSK_CP_, RS_CP_, NOX_CP_, | Patient-CBP | 24 | 1.5T | Heat | Right | Vs. Innocuous | No cue | MNI | Table 2 | 19 |
| ([Lopez-Sola et al., 2010b](#_ENREF_91)) | HC, MECH_HC_, RIGHT_HC_, REST_HC_, CUED_HC_ | Healthy Volunteer | 25 | 1.5T | Pressure | Right | Vs. rest | Cue | Tal | Table 1 | 19 |
| ([Lopez-Sola et al., 2010a](#_ENREF_90)) | HC, THER_HC_, RIGHT_HC_, INNOC_HC_, CUED_HC_ | Healthy Volunteer | 20 | 1.5T | Heat | Right | Vs. innocuous | Cue | MNI | Table 2 | 8 |
| ([Lui et al., 2008](#_ENREF_92)) | HC, MECH_HC_, RIGHT_HC_, REST_HC_, CUED_HC_ | Healthy Volunteer | 14 | 1.5T | Pressure | Right | Vs. rest | Cue | MNI | Table 1 | 20 |
| ([Lutz et al., 2013](#_ENREF_93)) | HC, THER_HC_, LEFT_HC_, INNOC_HC_, CUED_HC_ | Healthy Volunteer | 14 | 3.0T | Heat | Left | Vs. innocuous | Cue | Tal | Supplementary Table 1 | 10 |
| ([Maeda et al., 2011](#_ENREF_94)) | HC, MECH_HC_, RIGHT_HC_, REST_HC_, NCUED_HC_ | Healthy Volunteer | 12 | 1.5T | Pressure | Right | Vs. rest | No cue | Tal | Table 2 | 22 |
| ([Maihofner and Handwerker, 2005](#_ENREF_96)) | HC, THER_HC_, LEFT_HC_, INNOC_HC_, NCUED_HC_, HYPER_HC_ | Healthy Volunteer | 12 | 1.5T | Heat | Left | Vs. innocuous, | No cue | Tal | Table 1 | 11 |
|  | HYPER_MAC_ | Healthy Volunteer | 12 | 1.5T | Heat and pressure | Left | Hyperalgesia vs. normalgesia | No cue | Tal | Table 2 | 27 |
| ([Maihofner et al., 2004](#_ENREF_99)) | HYPER_HC_ | Healthy Volunteer | 11 | 1.5T | Pressure | Left | Vs. rest | No cue | Tal | Table 1 | 8 |
|  | HYPER_MAC_ | Healthy Volunteer | 11 | 1.5T | Pressure | Left | Hyperalgesia vs. normalgesia | No cue | Tal | Table 1 | 7 |
| ([Maihofner et al., 2005](#_ENREF_95)) | CP, NEUR_CP_, CS_CP_, ALDN_CP_, MECH_CP_ | Patient-CRPS | 12 | 1.5T | Pressure | Right | Vs. rest | No cue | Tal | Table 2 | 19 |
| ([Maihofner et al., 2006b](#_ENREF_98)) | HC, THER_HC_, MECH_HC_ RIGHT_HC_, REST_HC_, NCUED_HC_ | Healthy Volunteer | 14 | 1.5T | Heat and Pressure | Right | Vs. rest | No cue | Tal | Table 1 | 39 |
| ([Maihofner et al., 2006a](#_ENREF_97)) | CP, NEUR_CP_, CS_CP_, ALDN_CP_, MECH_CP_ | Patients-CRPS | 12 | 1.5T | Tactile | Left | Vs. rest | No cue | Tal | Supplementary Table 2 | 24 |
| ([Maihofner et al., 2011](#_ENREF_100)) | HC, MECH_HC_, LEFT_HC_, REST_HC_, NCUED_HC_ | Healthy Volunteer | 12 | 1.5T | Pressure | Left | Vs. rest | No cue | Tal | Table 1 | 18 |
| ([Mantini et al., 2009](#_ENREF_101)) | HC, ELEC_HC_, RIGHT_HC_, REST_HC_, NCUED_HC_ | Healthy Volunteer | 10 | 1.5T | Electrical | Right | Vs. rest | No cue | Tal | Supplementary Table 3 | 9 |
| ([Markl et al., 2013](#_ENREF_102)) | HC, ELEC_HC_, LEFT_HC_, REST_HC_, NCUED_HC_ | Healthy Volunteer | 15 | 1.5T | Electrical | Left | Vs. rest | No cue | MNI | Table 2 | 8 |
| ([Martin et al., 2013](#_ENREF_103)) | HC, THER_HC_, LEFT_HC_, INNOC_HC_, NCUED_HC_ | Healthy Volunteer | 23 | 3.0T | Heat | Left | Vs. innocuous | No cue | MNI | Table 2 | 6 |
| ([Misra and Coombes, 2014](#_ENREF_104)) | HC, THER_HC_, RIGHT_HC_, NCUED_HC_ | Healthy Volunteer | 15 | 3.0T | Heat | Right | - | No cue | MNI | Table 2 | 25 |
| ([Mobascher et al., 2009a](#_ENREF_107)) | HC, THER_HC_, LEFT_HC_, REST_HC_, NCUED_HC_ | Healthy Volunteer | 20 | 3.0T | Heat | Left | Vs. rest | No cue | MNI | Table 1 | 17 |
| ([Mobascher et al., 2009b](#_ENREF_108)) | HC, THER_HC_, LEFT_HC_, REST_HC_, NCUED_HC_ | Healthy Volunteer | 12 | 3.0T | Heat | Left | Vs. rest | No cue | MNI | Table 1 | 17 |
| ([Mobascher et al., 2010b](#_ENREF_106)) | HC, THER_HC_, LEFT_HC_, REST_HC_, NCUED_HC_ | Healthy Volunteer | 32 | 3.0T | Heat | Left | Vs. rest | No cue | MNI | Table 1 | 17 |
| ([Mobascher et al., 2010a](#_ENREF_105)) | HC, THER_HC_, LEFT_HC_, REST_HC_, NCUED_HC_ | Healthy Volunteer | 57 | 3.0T | Heat | Left | Vs. rest | No cue | MNI | Table 1 | 17 |
| ([Mochizuki et al., 2007](#_ENREF_109)) | HC, THER_HC_, LEFT_HC_, INNOC_HC_, NCUED_HC_ | Healthy Volunteer | 14 | 3.0T | Cold | Left | Vs. innocuous | No cue | MNI | Table 2 | 7 |
| ([Mohr et al., 2008](#_ENREF_110)) | HC, THER_HC_, RIGHT_HC_, INNOC_HC_, CUED_HC_ | Healthy Volunteer | 15 | 1.5T | Cold | Right | Vs. innocuous | Cue | MNI | Supplementary Table 3 | 19 |
|  | HYPER_HC_ | Healthy Volunteer | 15 | 1.5T | Cold | Right | Vs. innocuous | Cue | MNI | Supplementary Table 3 | 25 |
|  | HYPER_MAC_ | Healthy Volunteer | 15 | 1.5T | Cold | Right | Hyperalgesia vs. normalgesia | Cue | MNI | Table 2 | 10 |
| ([Morrison et al., 2004](#_ENREF_112)) | HC, MECH_HC_, LEFT_HC_, INNOC_HC_, CUED_HC_ | Healthy Volunteer | 14 | 1.5T | Pressure | Left | Vs. innocuous | Cue | Tal | Table 1 | 5 |
| ([Morrison and Downing, 2007](#_ENREF_111)) | HC, MECH_HC_, LEFT_HC_, INNOC_HC_, NCUED_HC_ | Healthy Volunteer | 11 | 1.5T | Pressure | Left | Vs. innocuous | No cue | Tal | Table 1 | 9 |
| ([Moulton et al., 2005](#_ENREF_114)) | HC, THER_HC_, LEFT_HC_, INNOC_HC_, NCUED_HC_ | Healthy Volunteer | 33 | 1.5T | Heat | Left | Vs. innocuous | No cue | Tal | Table 1 | 17 |
| ([Moulton et al., 2007](#_ENREF_116)) | HYPER_MAC_ | Healthy Volunteer | 12 | 3.0T | Heat | Left | Vs. innocuous | No cue | MNI | Table 1 | 9 |
| ([Moulton et al., 2011](#_ENREF_113)) | HC, THER_HC_, INNOC_HC_, NCUED_HC_ | Healthy Volunteer | 11 | 3.0T | Heat | Mixed | Vs. innocuous | No cue | MNI | Table 4 | 45 |
|  | CP, NEUR_CP_, RS_CP_, NOX_CP_, CP_HC_MAC_ | Patient-M | 11 | 3.0T | Heat | Mixed | Vs. innocuous, Patient vs. healthy volunteer | No cue | MNI | Table 3, Supplementary Table 3 | 34, 27 |
| ([Moulton et al., 2012](#_ENREF_115)) | HC, THER_HC_, RIGHT_HC_, REST_HC_, NCUED_HC_ | Healthy Volunteer | 12 | 3.0T | Heat | Right | Vs. rest | No cue | MNI | Table 1 | 11 |
| ([Naglatzki et al., 2012](#_ENREF_117)) | HC, ELEC_HC_, NCUED_HC_ | Healthy Volunteer | 13 | 1.5T | Electrical | - | - | No cue | MNI | Table 1 | 8 |
| ([Nickel et al., 2014](#_ENREF_118)) | HC, ELEC_HC_, RIGHT_HC_, NCUED_HC_ | Healthy Volunteer | 48 | 3.0T | Electrical | Right | - | No cue | Tal | Supplementary Table 1 | 25 |
| ([Nicotra et al., 2006](#_ENREF_119)) | HC, ELEC_HC_, RIGHT_HC_, REST_HC_, CUED_HC_ | Healthy Volunteer | 7 | 1.5T | Electrical | Right | Vs. rest, | Cue | Tal | Table 2 | 5 |
| ([Obermann et al., 2009](#_ENREF_120)) | HC, ELEC_HC_, RIGHT_HC_, REST_HC_, NCUED_HC_ | Healthy Volunteer | 11 | 1.5T | Electrical | Right | Vs. rest | No cue | MNI | Table 3 | 11 |
| ([Ochsner et al., 2006](#_ENREF_121)) | HC, THER_HC_, RIGHT_HC_, REST_HC_, NCUED_HC_ | Healthy Volunteer | 13 | 3.0T | Heat | Right | Vs. rest | No cue | MNI | Table 1 | 19 |
| ([Oshiro et al., 2007](#_ENREF_122)) | HC, THER_HC_, LEFT_HC_, INNOC_HC_, CUED_HC_ | Healthy Volunteer | 12 | 1.5T | Heat | Left | Vs. innocuous | Cue | MNI | Supplementary Table 1 | 15 |
| ([Oshiro et al., 2009](#_ENREF_123)) | HC, THER_HC_, LEFT_HC_, REST_HC_, CUED_HC_ | Healthy Volunteer | 12 | 1.5T | Heat | Left | Vs. rest | Cue | MNI | Supplementary Table 1 | 20 |
| ([Parks et al., 2011](#_ENREF_124)) | HC, MECH_HC_, REST_HC_, NCUED_HC_ | Healthy Volunteer | 9 | 3.0T | Pressure | Mixed | Vs. rest | No cue | MNI | Supplementary Table 3 | 26 |
|  | CP, MSK_CP_, CS_CP_, NOX_CP_ | Patient-OA | 14 | 3.0T | Pressure | Mixed | Vs. rest | No cue | MNI | Supplementary Table 3 | 22 |
| ([Peltz et al., 2011](#_ENREF_125)) | HC, THER_HC_, LEFT_HC_, INNOC_HC_, NCUED_HC_ | Healthy Volunteer | 11 | 3.0T | Heat and cold | Left | Vs. innocuous | No cue | Tal | Table 1 | 22, 18 |
| ([Perini et al., 2013](#_ENREF_126)) | HC, THER_HC_, LEFT_HC_, REST_HC_, CUED_HC_ | Healthy Volunteer | 18 | 3.0T | Heat and Cold | Left | Vs. rest | Cue | Tal | Table 1 | 7 |
| ([Peyron et al., 2004](#_ENREF_127)) | CP, NEUR_CP_, CS_CP_, ALDN_CP_, MECH_CP_ | Patient-NP | 27 | 1.0T | Pressure | Left | Vs. rest | No cue | Tal | Supplementary Table 1 | 14 |
| ([Piche et al., 2010](#_ENREF_128)) | HC, ELEC_HC_, RIGHT_HC_, REST_HC_, NCUED_HC_ | Healthy Volunteer | 11 | 3.0T | Electrical | Right | Vs. rest | No cue | MNI | Table 1, Supplementary Table 2 | 23, 21 |
| ([Ploner et al., 2010](#_ENREF_129)) | HC, THER_HC_, RIGHT_HC_, INNOC_HC_, NCUED_HC_ | Healthy Volunteer | 16 | 3.0T | Heat | Right | Vs. innocuous | No cue | MNI | Table 1 | 17 |
| ([Pomares et al., 2013](#_ENREF_130)) | HC, THER_HC_, LEFT_HC_, REST_HC_, NCUED_HC_ | Healthy Volunteer | 21 | 3.0T | Heat | Left | Vs. rest | No cue | MNI | Table 1 | 34 |
| ([Pujol et al., 2009](#_ENREF_131)) | HC, MECH_HC_, RIGHT_HC_, REST_HC_, NCUED_HC_ | Healthy Volunteer | 9 | 1.5T | Pressure | Right | Vs. rest | No cue | Tal | Table 1, 4 | 6, 12 |
|  | CP, FM_CP_, RS_CP_, NOX_CP_, MECH_CP_, CP_HC_MAC_ | Patient-FM | 9 | 1.5T | Pressure | Right | Vs. rest, Patient vs. healthy volunteer | No cue | Tal | Table 1, 4 | 14, 12, 9 |
| ([Pukall et al., 2005](#_ENREF_132)) | CP, NEUR_CP_, CS_CP_, ALDN_CP_, MECH_CP_ | Patient-VVS | 14 | 1.5T | Pressure | Central | Vs. rest | No cue | Tal | Table 3 | 15 |
| ([Qiu et al., 2006](#_ENREF_133)) | HC, THER_HC_, RIGHT_HC_, REST_HC_, NCUED_HC_ | Healthy Volunteer | 13 | 3.0T | Heat | Right | Vs. rest | No cue | MNI | Table 1, 2 | 10 |
| ([Raij et al., 2005](#_ENREF_134)) | HC, THER_HC_, LEFT_HC_, REST_HC_, NCUED_HC_ | Healthy Volunteer | 14 | 3.0T | Heat | Left | Vs. rest | No cue | Tal | Supplementary Table 3 | 11 |
| ([Remy et al., 2003](#_ENREF_135)) | HC, THER_HC_, LEFT_HC_, INNOC_HC_, NCUED_HC_ | Healthy Volunteer | 12 | 3.0T | Heat | Left | Vs. innocuous | No cue | MNI | Table 1 | 7 |
| ([Roberts et al., 2008](#_ENREF_136)) | HC, THER_HC_, LEFT_HC_, INNOC_HC_, NCUED_HC_ | Healthy Volunteer | 10 | 1.5T | Heat | Left | Vs. innocuous | No cue | Tal | Table 2 | 17 |
| ([Rolls et al., 2003](#_ENREF_137)) | HC, MECH_HC_, LEFT_HC_, REST_HC_, NCUED_HC_ | Healthy Volunteer | 9 | 3.0T | Pressure | Left | Vs. rest | No cue | MNI | In text | 15 |
| ([Roy et al., 2009](#_ENREF_138)) | HC, ELEC_HC_, RIGHT_HC_, REST_HC_, NCUED_HC_ | Healthy Volunteer | 12 | 3.0T | Electrical | Right | Vs. rest | No cue | MNI | Supplementary Table 1 | 29 |
| ([Russo et al., 2012](#_ENREF_139)) | HC, THER_HC_, REST_HC_, NCUED_HC_ | Healthy Volunteer | 16 | 3.0T | Heat | Mixed | Vs. rest | No cue | Tal | Table 3 | 14 |
|  | CP, NEUR_CP_, RS_CP_, NOX_CP_, CP_MAC_ | Patient-M | 16 | 3.0T | Heat | Mixed | Vs. rest, Patient vs. Healthy volunteer | No cue | Tal | Table 2 | 16, 1 |
| ([Schoedel et al., 2008](#_ENREF_140)) | HC, MECH_HC_, LEFT_HC_, REST_HC_, NCUED_HC_ | Healthy Volunteer | 11 | 1.5T | Pressure | Left | Vs. rest | No cue | Tal | Table 1 | 14 |
| ([Schweinhardt et al., 2006](#_ENREF_141)) | CP, NEUR_CP_, CS_CP_, ALDN_CP_ | Patient-NP | 8 | 1.5T | Pressure | Mixed | Vs. rest | No cue | MNI | Table 3 | 18, 17 |
| ([Seifert and Maihofner, 2007](#_ENREF_144)) | HC, THER_HC_, RIGHT_HC_, INNOC_HC_, NCUED_HC_, HYPER_HC_, HYPER_MAC_ | Healthy Volunteer | 12 | 1.5T | Cold | Right | Vs. innocuous, Hyperalgesia vs. normalgesia | No cue | Tal | Table 1, 2 | 19, 21, 12 |
| ([Seifert et al., 2008](#_ENREF_143)) | HC, THER_HC_, MECH_HC_, RIGHT_HC_, INNOC_HC_, NCUED_HC_, HYPER_HC_, HYPER_MAC_ | Healthy Volunteer | 14 | 1.5T | Heat and Pressure | Right | Vs. innocuous, Hyperalgesia vs. normalgesia | No cue | Tal | Table 1 | 6, 19, 21 |
| ([Seifert et al., 2009](#_ENREF_142)) | HC, MECH_HC_, RIGHT_HC_, REST_HC_, NCUED_HC_, HYPER_HC_ | Healthy Volunteer | 12 | 1.5T | Pressure | Right | Vs. rest | No cue | Tal | Table 1 | 6, 19 |
| ([Seifert et al., 2013](#_ENREF_145)) | HC, THER_HC_, LEFT_HC_, INNOC_HC_, NCUED_HC_ | Healthy Volunteer | 9 | 1.5T | Heat | Left | Vs. innocuous | No cue | Tal | Supplementary Table 1.1 | 28 |
| ([Seminowicz and Davis, 2006](#_ENREF_146)) | HC, ELEC_HC_, LEFT_HC_, REST_HC_, NCUED_HC_ | Healthy Volunteer | 22 | 1.5T | Electrical | Left | Vs. rest | No cue | Tal | Table 1 | 12 |
| ([Shelton et al., 2012](#_ENREF_147)) | HC, THER_HC_, LEFT_HC_, INNOC_HC_, NCUED_HC_ | Healthy Volunteer | 11 | 3.0T | Heat | Left | Vs. innocuous | No cue | MNI | Table 5 | 30 |
| ([Shenoy et al., 2011](#_ENREF_148)) | HC, THER_HC_, LEFT_HC_, REST_HC_, NCUED_HC_, HYPER_HC_, | Healthy Volunteer | 12 | 1.5T | Heat | Left | Vs. rest | No cue | Tal | Table 1 | 14, 11 |
| ([Shukla et al., 2011](#_ENREF_149)) | HC, THER_HC_, LEFT_HC_, INNOC_HC_, NCUED_HC_, | Healthy Volunteer | 10 | 3.0T | Heat | Left | Vs. innocuous | No cue | Tal | Table 1 | 6 |
| ([Smith et al., 2002](#_ENREF_150)) | HC, THER_HC_, LEFT_HC_, INNOC_HC_, NCUED_HC_ | Healthy Volunteer | 8 | 3.0T | Heat | Left | Vs. innocuous | No cue | MNI | Table 2 | 13 |
| ([Sofina et al., 2014](#_ENREF_151)) | HC, THER_HC_, NCUED_HC_ | Healthy Volunteer | 4 | 3.0T | Heat | - | - | No cue | MNI | Table 1 | 16 |
| ([Sprenger et al., 2011](#_ENREF_152)) | HC, THER_HC_, LEFT_HC_, REST_HC_, NCUED_HC_ | Healthy Volunteer | 22 | 3.0T | Heat | Left | Vs. rest | No cue | MNI | Supplementary Table 2 | 35 |
| ([Stammler et al., 2008](#_ENREF_153)) | HC, MECH_HC_, RIGHT_HC_, REST_HC_, NCUED_HC_, HYPER_HC_ | Healthy Volunteer | 12 | 1.5T | Pressure | Right | Vs. rest | No cue | Tal | Table 1 | 17, 28 |
| ([Starr et al., 2009](#_ENREF_154)) | HC, THER_HC_, LEFT_HC_, RIGHT_HC_, REST_HC_, NCUED_HC_ | Healthy Volunteer | 13 | 1.5T | Heat | Mixed | Vs. rest | No cue | MNI | Table 2 | 40 |
| ([Straube et al., 2009](#_ENREF_155)) | HC, ELEC_HC_, LEFT_HC_, REST_HC_, NCUED_HC_ | Healthy Volunteer | 24 | 1.5T | Electrical | Left | Vs. rest | No cue | Tal | Table 2 | 16 |
| ([Strigo et al., 2003](#_ENREF_156)) | HC, THER_HC_, INNOC_HC_, NCUED_HC_ | Healthy Volunteer | 7 | 1.5T | Heat | Midline | Vs. innocuous | No cue | Tal | Table 2 | 25 |
| ([Symonds et al., 2006](#_ENREF_157)) | HC, ELEC_HC_, LEFT_HC_, RIGHT_HC_, REST_HC_ | Healthy Volunteer | 9 | 3.0T | Electrical | Mixed | Vs. rest | - | Tal | Table 1 | 15 |
| ([Takahashi et al., 2011](#_ENREF_158)) | HC, ELEC_HC_, LEFT_HC_, REST_HC_, NCUED_HC_ | Healthy Volunteer | 13 | 3.0T | Electrical | Left | Vs. rest | No cue | MNI | Table 3 | 16 |
| ([Ter Minassian et al., 2013](#_ENREF_159)) | HC, ELEC_HC_, RIGHT_HC_, INNOC_HC_, CUED_HC_ | Healthy Volunteer | 20 | 1.5T | Electrical | Right | Vs. innocuous | Cue | MNI | Supplementary Table 3 | 73 |
| ([Theysohn et al., 2014](#_ENREF_160)) | HC, ELEC_HC_, LEFT_HC_, NCUED_HC_ | Healthy Volunteer | 14 | 1.5T | Electrical | Left | - | No cue | MNI | Table 1 | 10 |
| ([Tracey et al., 2000](#_ENREF_161)) | HC, THER_HC_, LEFT_HC_, INNOC_HC_, NCUED_HC_ | Healthy Volunteer | 6 | 1.5T | Heat, Cold | Left | Vs. innocuous | No cue | Tal | Table 1 | 25 |
| ([Tseng et al., 2010](#_ENREF_163)) | HC, THER_HC_, RIGHT_HC_, INNOC_HC_, NCUED_HC_ | Healthy Volunteer | 12 | 3.0T | Heat | Right | Vs. innocuous | No cue | MNI | Table 2 | 31 |
| ([Tseng et al., 2013](#_ENREF_162)) | HC, THER_HC_, RIGHT_HC_, INNOC_HC_, NCUED_HC_ | Healthy Volunteer | 11 | 3.0T | Heat | Right | Vs. innocuous | No cue | MNI | Supplementary Table 2 | 7 |
|  | CP, NEUR_CP_, CS_CP_, ALDN_CP_ | Patient-DN | 11 | 3.0T | Heat | Right | Vs. innocuous | No cue | MNI | Supplementary Table 2 | 16 |
| ([Uematsu et al., 2011](#_ENREF_164)) | HC, MECH_HC_, RIGHT_HC_, REST_HC_, NCUED_HC_ | Healthy Volunteer | 17 | 1.5T | Pressure | Right | Vs. rest | No cue | MNI | Table 2 | 31 |
| ([Vachon-Presseau et al., 2013](#_ENREF_165)) | HC, THER_HC_, INNOC_HC_, NCUED_HC_ | Healthy Volunteer | 18 | 3.0T | Heat | - | Vs. innocuous | No cue | MNI | Supplementary Table 1 | 17 |
|  | CP, MSK_CP_, RS_CP_, NOX_CP_ | Patient-CBP | 16 | 3.0T | Heat | - | Vs. innocuous | No cue | MNI | Supplementary Table 1 | 15 |
| ([Valet et al., 2004](#_ENREF_166)) | HC, THER_HC_, RIGHT_HC_, INNOC_HC_, NCUED_HC_ | Healthy Volunteer | 7 | 1.5T | Heat | Right | Vs. innocuous | No cue | MNI | Table 1 | 16 |
| ([Vanhaudenhuyse et al., 2009](#_ENREF_167)) | HC, THER_HC_, LEFT_HC_, REST_HC_, NCUED_HC_ | Healthy Volunteer | 13 | 3.0T | Heat | Left | Vs. rest | No cue | MNI | Table 2 | 10 |
| ([Vartiainen et al., 2009](#_ENREF_168)) | HC, THER_HC_, INNOC_HC_, NCUED_HC_ | Healthy Volunteer | 9 | 3.0T | Heat | Mixed | Vs. innocuous | No cue | MNI | Table 2 | 15 |
|  | CP, NEUR_CP_, RS_CP_, NOX_CP_ | Patient-PHN | 8 | 3.0T | Heat | Mixed | Vs. innocuous, Patient vs. healthy volunteer | No cue | MNI | Supplementary Table 2 and Table 3 | 3, 3 |
| ([Veldhuijzen et al., 2009](#_ENREF_169)) | HC, THER_HC_, LEFT_HC_, REST_HC_, NCUED_HC_ | Healthy Volunteer | 10 | 3.0T | Heat | Left | Vs. rest | No cue | Tal | Table 2 | 20 |
| ([Villemure and Bushnell, 2009](#_ENREF_170)) | LM_HC_ | Healthy Volunteer | 14 | 1.5T | Heat | Left | Vs. innocuous | No cue | MNI | Table 3 | 5 |
| ([Wager et al., 2004](#_ENREF_171)) | HC, THER_HC_, LEFT_HC_, REST_HC_, CUED_HC_ | Healthy Volunteer | 23 | 3.0T | Heat | Left | Vs. rest | Cue | MNI | Supplementary Table 1 | 26 |
| ([Wagner et al., 2009](#_ENREF_172)) | LM_HC_ | Healthy Volunteer | 40 | 1.5T | Heat | Mixed | - | No Cue | MNI |  |  |
| ([Wang et al., 2011](#_ENREF_173)) | HC, MECH_HC_, LEFT_HC_, REST_HC_, CUED_HC_ | Healthy Volunteer | 8 | 3.0T | Pressure | Left | Vs. rest | Cue | Tal | Table 1 | 18 |
| ([Wiech et al., 2006](#_ENREF_175)) | HC, ELEC_HC_, LEFT_HC_, REST_HC_, CUED_HC_ | Healthy Volunteer | 12 | 3.0T | Electrical | Left | Vs. rest | Cue | MNI | Table 1 | 25 |
| ([Wiech et al., 2010](#_ENREF_176)) | HC, ELEC_HC_, RIGHT_HC_, REST_HC_, CUED_HC_ | Healthy Volunteer | 16 | 3.0T | Heat | Right | Vs. rest | Cue | MNI | Supplementary Table 3 | 25 |
| ([Wey et al., 2014](#_ENREF_174)) | HC, MECH_HC_, LEFT_HC_, NCUED_HC_ | Healthy Volunteer | 11 | 3.0T | Pressure | Left | - | No cue | MNI | Table 1 | 9 |
| ([Zambreanu et al., 2005](#_ENREF_177)) | HYPER_MAC_ | Healthy Volunteer | 12 | 3.0T | Pressure | Right | Vs. innocuous | No cue | MNI | Table 1 | 15 |
| ([Ziv et al., 2010](#_ENREF_178)) | HC, THER_HC_, INNOC_HC_, CUED_HC_ | Healthy Volunteer | 10 | 3.0T | Heat | Mixed | Vs. innocuous | Cue | Tal | Table 1 | 15 |

**References**

ALBANESE, M. C., DUERDEN, E. G., RAINVILLE, P. & DUNCAN, G. H. 2007. Memory traces of pain in human cortex. *J Neurosci,* 27**,** 4612-20.

ALBUQUERQUE, R. J., DE LEEUW, R., CARLSON, C. R., OKESON, J. P., MILLER, C. S. & ANDERSEN, A. H. 2006. Cerebral activation during thermal stimulation of patients who have burning mouth disorder: an fMRI study. *Pain,* 122**,** 223-34.

APKARIAN, A. V., GELNAR, P. A., KRAUSS, B. R. & SZEVERENYI, N. M. 2000. Cortical responses to thermal pain depend on stimulus size: a functional MRI study. *J Neurophysiol,* 83**,** 3113-22.

ATLAS, L. Y., BOLGER, N., LINDQUIST, M. A. & WAGER, T. D. 2010. Brain mediators of predictive cue effects on perceived pain. *J Neurosci,* 30**,** 12964-77.

BALIKI, M. N., CHIALVO, D. R., GEHA, P. Y., LEVY, R. M., HARDEN, R. N., PARRISH, T. B. & APKARIAN, A. V. 2006. Chronic pain and the emotional brain: specific brain activity associated with spontaneous fluctuations of intensity of chronic back pain. *J Neurosci,* 26**,** 12165-73.

BALIKI, M. N., GEHA, P. Y., FIELDS, H. L. & APKARIAN, A. V. 2010. Predicting value of pain and analgesia: nucleus accumbens response to noxious stimuli changes in the presence of chronic pain. *Neuron,* 66**,** 149-60.

BALIKI, M. N., GEHA, P. Y., JABAKHANJI, R., HARDEN, N., SCHNITZER, T. J. & APKARIAN, A. V. 2008. A preliminary fMRI study of analgesic treatment in chronic back pain and knee osteoarthritis. *Molecular pain,* 4**,** 47.

BECERRA, L., BREITER, H. C., WISE, R., GONZALEZ, R. G. & BORSOOK, D. 2001. Reward circuitry activation by noxious thermal stimuli. *Neuron,* 32**,** 927-46.

BECERRA, L., MORRIS, S., BAZES, S., GOSTIC, R., SHERMAN, S., GOSTIC, J., PENDSE, G., MOULTON, E., SCRIVANI, S., KEITH, D., CHIZH, B. & BORSOOK, D. 2006. Trigeminal neuropathic pain alters responses in CNS circuits to mechanical (brush) and thermal (cold and heat) stimuli. *J Neurosci,* 26**,** 10646-57.

BECERRA, L. R., BREITER, H. C., STOJANOVIC, M., FISHMAN, S., EDWARDS, A., COMITE, A. R., GONZALEZ, R. G. & BORSOOK, D. 1999. Human brain activation under controlled thermal stimulation and habituation to noxious heat: an fMRI study. *Magn Reson Med,* 41**,** 1044-57.

BERNA, C., LEKNES, S., HOLMES, E. A., EDWARDS, R. R., GOODWIN, G. M. & TRACEY, I. 2010. Induction of depressed mood disrupts emotion regulation neurocircuitry and enhances pain unpleasantness. *Biol Psychiatry,* 67**,** 1083-90.

BINGEL, U., LORENZ, J., SCHOELL, E., WEILLER, C. & BUCHEL, C. 2006. Mechanisms of placebo analgesia: rACC recruitment of a subcortical antinociceptive network. *Pain,* 120**,** 8-15.

BINGEL, U., QUANTE, M., KNAB, R., BROMM, B., WEILLER, C. & BUCHEL, C. 2002. Subcortical structures involved in pain processing: evidence from single-trial fMRI. *Pain,* 99**,** 313-21.

BINGEL, U., QUANTE, M., KNAB, R., BROMM, B., WEILLER, C. & BUCHEL, C. 2003. Single trial fMRI reveals significant contralateral bias in responses to laser pain within thalamus and somatosensory cortices. *Neuroimage,* 18**,** 740-8.

BINGEL, U., ROSE, M., GLASCHER, J. & BUCHEL, C. 2007a. fMRI reveals how pain modulates visual object processing in the ventral visual stream. *Neuron,* 55**,** 157-67.

BINGEL, U., SCHOELL, E., HERKEN, W., BUCHEL, C. & MAY, A. 2007b. Habituation to painful stimulation involves the antinociceptive system. *Pain,* 131**,** 21-30.

BINGEL, U., WANIGASEKERA, V., WIECH, K., NI MHUIRCHEARTAIGH, R., LEE, M. C., PLONER, M. & TRACEY, I. 2011. The effect of treatment expectation on drug efficacy: imaging the analgesic benefit of the opioid remifentanil. *Sci Transl Med,* 3**,** 70ra14.

BOGDANOV, V. B., VIGANO, A., NOIRHOMME, Q., BOGDANOVA, O. V., GUY, N., LAUREYS, S., RENSHAW, P. F., DALLEL, R., PHILLIPS, C. & SCHOENEN, J. 2015. Cerebral responses and role of the prefrontal cortex in conditioned pain modulation: an fMRI study in healthy subjects. *Behav Brain Res,* 281**,** 187-98.

BOLY, M., BALTEAU, E., SCHNAKERS, C., DEGUELDRE, C., MOONEN, G., LUXEN, A., PHILLIPS, C., PEIGNEUX, P., MAQUET, P. & LAUREYS, S. 2007. Baseline brain activity fluctuations predict somatosensory perception in humans. *Proc Natl Acad Sci U S A,* 104**,** 12187-92.

BORNHOVD, K., QUANTE, M., GLAUCHE, V., BROMM, B., WEILLER, C. & BUCHEL, C. 2002. Painful stimuli evoke different stimulus-response functions in the amygdala, prefrontal, insula and somatosensory cortex: a single-trial fMRI study. *Brain,* 125**,** 1326-36.

BOTVINICK, M., JHA, A. P., BYLSMA, L. M., FABIAN, S. A., SOLOMON, P. E. & PRKACHIN, K. M. 2005. Viewing facial expressions of pain engages cortical areas involved in the direct experience of pain. *Neuroimage,* 25**,** 312-9.

BRINKMEYER, J., MOBASCHER, A., WARBRICK, T., MUSSO, F., WITTSACK, H. J., SALEH, A., SCHNITZLER, A. & WINTERER, G. 2010. Dynamic EEG-informed fMRI modeling of the pain matrix using 20-ms root mean square segments. *Hum Brain Mapp,* 31**,** 1702-12.

BROOKS, J. C., ZAMBREANU, L., GODINEZ, A., CRAIG, A. D. & TRACEY, I. 2005. Somatotopic organisation of the human insula to painful heat studied with high resolution functional imaging. *Neuroimage,* 27**,** 201-9.

BROOKS, J. C. W., BIMSON, W. E., ROBERTS, N. & NURMIKKO, T. J. 2003. Functional Magnetic Resonance Imaging of capsaicin-induced thermal hyperalgesia. *Proceedings of the 10th World Congress on Pain.*

BROOKS, J. C. W., NURMIKKO, T. J., BIMSON, W. E., SINGH, K. D. & ROBERTS, N. 2002. fMRI of thermal pain: effects of stimulus laterality and attention. *NeuroImage,* 15**,** 293-301.

BUCHEL, C., BORNHOVD, K., QUANTE, M., GLAUCHE, V., BROMM, B. & WEILLER, C. 2002. Dissociable neural responses related to pain intensity, stimulus intensity, and stimulus awareness within the anterior cingulate cortex: a parametric single-trial laser functional magnetic resonance imaging study. *J Neurosci,* 22**,** 970-6.

CARLSSON, K., ANDERSSON, J., PETROVIC, P., PETERSSON, K. M., OHMAN, A. & INGVAR, M. 2006. Predictability modulates the affective and sensory-discriminative neural processing of pain. *Neuroimage,* 32**,** 1804-14.

CHRISTMANN, C., KOEPPE, C., BRAUS, D. F., RUF, M. & FLOR, H. 2007. A simultaneous EEG-fMRI study of painful electric stimulation. *Neuroimage,* 34**,** 1428-37.

COGHILL, R. C., MCHAFFIE, J. G. & YEN, Y. F. 2003. Neural correlates of interindividual differences in the subjective experience of pain. *Proc Natl Acad Sci U S A,* 100**,** 8538-42.

COLE, L. J., FARRELL, M. J., DUFF, E. P., BARBER, J. B., EGAN, G. F. & GIBSON, S. J. 2006. Pain sensitivity and fMRI pain-related brain activity in Alzheimer's disease. *Brain,* 129**,** 2957-65.

COLE, L. J., FARRELL, M. J., GIBSON, S. J. & EGAN, G. F. 2010. Age-related differences in pain sensitivity and regional brain activity evoked by noxious pressure. *Neurobiol Aging,* 31**,** 494-503.

COOK, D. B., LANGE, G., CICCONE, D. S., LIU, W. C., STEFFENER, J. & NATELSON, B. H. 2004. Functional imaging of pain in patients with primary fibromyalgia. *J Rheumatol,* 31**,** 364-78.

CORRADI-DELL'ACQUA, C., HOFSTETTER, C. & VUILLEUMIER, P. 2011. Felt and seen pain evoke the same local patterns of cortical activity in insular and cingulate cortex. *J Neurosci,* 31**,** 17996-8006.

DAVIS, K. D., POPE, G. E., CRAWLEY, A. P. & MIKULIS, D. J. 2002. Neural correlates of prickle sensation: a percept-related fMRI study. *Nat Neurosci,* 5**,** 1121-2.

DE LA FUENTE-SANDOVAL, C., FAVILA, R., GOMEZ-MARTIN, D., LEON-ORTIZ, P. & GRAFF-GUERRERO, A. 2012. Neural response to experimental heat pain in stable patients with schizophrenia. *J Psychiatr Res,* 46**,** 128-34.

DE LA FUENTE-SANDOVAL, C., FAVILA, R., GOMEZ-MARTIN, D., PELLICER, F. & GRAFF-GUERRERO, A. 2010. Functional magnetic resonance imaging response to experimental pain in drug-free patients with schizophrenia. *Psychiatry Res,* 183**,** 99-104.

DE LEEUW, R., DAVIS, C. E., ALBUQUERQUE, R., CARLSON, C. R. & ANDERSEN, A. H. 2006. Brain activity during stimulation of the trigeminal nerve with noxious heat. *Oral Surg Oral Med Oral Pathol Oral Radiol Endod,* 102**,** 750-7.

DERBYSHIRE, S. W. & OSBORN, J. 2009. Offset analgesia is mediated by activation in the region of the periaqueductal grey and rostral ventromedial medulla. *Neuroimage,* 47**,** 1002-6.

DERBYSHIRE, S. W. G., WHALLEY, M. G., STENGER, V. A. & OAKLEY, D. A. 2004. Cerebral activation during hypnotically induced and imagined pain. *NeuroImage,* 23**,** 392-401.

DIERS, M., YILMAZ, P., RANCE, M., THIEME, K., GRACELY, R. H., ROLKO, C., SCHLEY, M. T., KIESSLING, U., WANG, H. & FLOR, H. 2012. Treatment-related changes in brain activation in patients with fibromyalgia syndrome. *Exp Brain Res,* 218**,** 619-28.

DOWNAR, J., MIKULIS, D. J. & DAVIS, K. D. 2003. Neural correlates of the prolonged salience of painful stimulation. *Neuroimage,* 20**,** 1540-51.

DUBE, A. A., DUQUETTE, M., ROY, M., LEPORE, F., DUNCAN, G. & RAINVILLE, P. 2009. Brain activity associated with the electrodermal reactivity to acute heat pain. *Neuroimage,* 45**,** 169-80.

DUCREUX, D., ATTAL, N., PARKER, F. & BOUHASSIRA, D. 2006. Mechanisms of central neuropathic pain: a combined psychophysical and fMRI study in syringomyelia. *Brain,* 129**,** 963-76.

DUNCKLEY, P., WISE, R. G., AZIZ, Q., PAINTER, D., BROOKS, J., TRACEY, I. & CHANG, L. 2005. Cortical processing of visceral and somatic stimulation: differentiating pain intensity from unpleasantness. *Neuroscience,* 133**,** 533-42.

FAIRHURST, M., WIECH, K., DUNCKLEY, P. & TRACEY, I. 2007. Anticipatory brainstem activity predicts neural processing of pain in humans. *Pain,* 128**,** 101-10.

FERRARO, S., GRAZZI, L., MANDELLI, M. L., AQUINO, D., DI FIORE, D., USAI, S., BRUZZONE, M. G., DI SALLE, F., BUSSONE, G. & CHIAPPARINI, L. 2012. Pain processing in medication overuse headache: a functional magnetic resonance imaging (fMRI) study. *Pain Med,* 13**,** 255-62.

FERRETTI, A., BABILONI, C., GRATTA, C. D., CAULO, M., TARTARO, A., BONOMO, L., ROSSINI, P. M. & ROMANI, G. L. 2003. Functional topography of the secondary somatosensory cortex for nonpainful and painful stimuli: an fMRI study. *Neuroimage,* 20**,** 1625-38.

FORKMANN, K., WIECH, K., RITTER, C., SOMMER, T., ROSE, M. & BINGEL, U. 2013. Pain-specific modulation of hippocampal activity and functional connectivity during visual encoding. *J Neurosci,* 33**,** 2571-81.

FRANKENSTEIN, U. N., RICHTER, W., MCINTYRE, M. C. & REMY, F. 2001. Distraction modulates anterior cingulate gyrus activations during the cold pressor test. *Neuroimage,* 14**,** 827-36.

FREUND, W., KLUG, R., WEBER, F., STUBER, G., SCHMITZ, B. & WUNDERLICH, A. P. 2009. Perception and suppression of thermally induced pain: a fMRI study. *Somatosens Mot Res,* 26**,** 1-10.

FREUND, W., STUBER, G., WUNDERLICH, A. P. & SCHMITZ, B. 2007. Cortical correlates of perception and suppression of electrically induced pain. *Somatosens Mot Res,* 24**,** 203-12.

FREUND, W., WUNDERLICH, A. P., STUBER, G., MAYER, F., STEFFEN, P., MENTZEL, M., SCHMITZ, B. & WEBER, F. 2011. The role of periaqueductal gray and cingulate cortex during suppression of pain in complex regional pain syndrome. *Clin J Pain,* 27**,** 796-804.

GARD, T., HOLZEL, B. K., SACK, A. T., HEMPEL, H., LAZAR, S. W., VAITL, D. & OTT, U. 2012. Pain Attenuation through Mindfulness is Associated with Decreased Cognitive Control and Increased Sensory Processing in the Brain. *Cerebral Cortex,* 22**,** 2692-2702.

GEHA, P. Y., BALIKI, M. N., WANG, X., HARDEN, R. N., PAICE, J. A. & APKARIAN, A. V. 2008. Brain dynamics for perception of tactile allodynia (touch-induced pain) in postherpetic neuralgia. *Pain,* 138**,** 641-56.

GELNAR, P. A., KRAUSS, B. R., SHEEHE, P. R., SZEVERENYI, N. M. & APKARIAN, A. V. 1999. A comparative fMRI study of cortical representations for thermal painful, vibrotactile, and motor performance tasks. *Neuroimage,* 10**,** 460-82.

GEUZE, E., WESTENBERG, H. G., JOCHIMS, A., DE KLOET, C. S., BOHUS, M., VERMETTEN, E. & SCHMAHL, C. 2007. Altered pain processing in veterans with posttraumatic stress disorder. *Arch Gen Psychiatry,* 64**,** 76-85.

GIESECKE, T., GRACELY, R. H., CLAUW, D. J., NACHEMSON, A., DUCK, M. H., SABATOWSKI, R., GERBERSHAGEN, H. J., WILLIAMS, D. A. & PETZKE, F. 2006. [Central pain processing in chronic low back pain. Evidence for reduced pain inhibition]. *Schmerz,* 20**,** 411-4, 416-7.

GODINHO, F., FAILLENOT, I., PERCHET, C., FROT, M., MAGNIN, M. & GARCIA-LARREA, L. 2012. How the pain of others enhances our pain: searching the cerebral correlates of 'compassional hyperalgesia'. *Eur J Pain,* 16**,** 748-59.

GOPINATH, K., GANDHI, P., GOYAL, A., JIANG, L., FANG, Y., OUYANG, L., GANJI, S., BUHNER, D., RINGE, W., SPENCE, J., BIGGS, M., BRIGGS, R. & HALEY, R. 2012. FMRI reveals abnormal central processing of sensory and pain stimuli in ill Gulf War veterans. *Neurotoxicology,* 33**,** 261-71.

GRACELY, R. H., PETZKE, F., WOLF, J. M. & CLAUW, D. J. 2002. Functional magnetic resonance imaging evidence of augmented pain processing in fibromyalgia. *Arthritis Rheum,* 46**,** 1333-43.

GRANT, J. A., COURTEMANCHE, J. & RAINVILLE, P. 2011. A non-elaborative mental stance and decoupling of executive and pain-related cortices predicts low pain sensitivity in Zen meditators. *Pain,* 152**,** 150-6.

GUNDEL, H., VALET, M., SORG, C., HUBER, D., ZIMMER, C., SPRENGER, T. & TOLLE, T. R. 2008. Altered cerebral response to noxious heat stimulation in patients with somatoform pain disorder. *Pain,* 137**,** 413-21.

HERDE, L., FORSTER, C., STRUPF, M. & HANDWERKER, H. O. 2007. Itch induced by a novel method leads to limbic deactivations a functional MRI study. *Journal of neurophysiology,* 98**,** 2347-56.

HIRAMATSU, T., NAKANISHI, K., YOSHIMURA, S., YOSHINO, A., ADACHI, N., OKAMOTO, Y., YAMAWAKI, S. & OCHI, M. 2014. The dorsolateral prefrontal network is involved in pain perception in knee osteoarthritis patients. *Neuroscience Letters,* 581**,** 109-114.

HOHMEISTER, J., KROLL, A., WOLLGARTEN-HADAMEK, I., ZOHSEL, K., DEMIRAKCA, S., FLOR, H. & HERMANN, C. 2010. Cerebral processing of pain in school-aged children with neonatal nociceptive input: an exploratory fMRI study. *Pain,* 150**,** 257-67.

IANNILLI, E., DEL GRATTA, C., GERBER, J. C., ROMANI, G. L. & HUMMEL, T. 2008. Trigeminal activation using chemical, electrical, and mechanical stimuli. *Pain,* 139**,** 376-88.

IBINSON, J. W. & VOGT, K. M. 2013. Pain does not follow the boxcar model: temporal dynamics of the BOLD fMRI signal during constant current painful electric nerve stimulation. *J Pain,* 14**,** 1611-9.

JANTSCH, H. H., KEMPPAINEN, P., RINGLER, R., HANDWERKER, H. O. & FORSTER, C. 2005. Cortical representation of experimental tooth pain in humans. *Pain,* 118**,** 390-9.

JENSEN, K. B., KOSEK, E., WICKSELL, R., KEMANI, M., OLSSON, G., MERLE, J. V., KADETOFF, D. & INGVAR, M. 2012. Cognitive Behavioral Therapy increases pain-evoked activation of the prefrontal cortex in patients with fibromyalgia. *Pain,* 153**,** 1495-503.

JENSEN, K. B., PETZKE, F., CARVILLE, S., FRANSSON, P., MARCUS, H., WILLIAMS, S. C., CHOY, E., MAINGUY, Y., GRACELY, R., INGVAR, M. & KOSEK, E. 2010. Anxiety and depressive symptoms in fibromyalgia are related to poor perception of health but not to pain sensitivity or cerebral processing of pain. *Arthritis Rheum,* 62**,** 3488-95.

KAKEDA, T., OGINO, Y., MORIYA, F. & SAITO, S. 2010. Sweet taste-induced analgesia: an fMRI study. *Neuroreport,* 21**,** 427-31.

KAMPING, S., BOMBA, I. C., KANSKE, P., DIESCH, E. & FLOR, H. 2013. Deficient modulation of pain by a positive emotional context in fibromyalgia patients. *Pain,* 154**,** 1846-55.

KIM, S. H., CHANG, Y., KIM, J. H., SONG, H. J., SEO, J., KIM, S. H., HAN, S. W., NAM, E. J., CHOI, T. Y., LEE, S. J. & KIM, S. K. 2011. Insular cortex is a trait marker for pain processing in fibromyalgia syndrome--blood oxygenation level-dependent functional magnetic resonance imaging study in Korea. *Clin Exp Rheumatol,* 29**,** S19-27.

KIM, S. H., LEE, Y., LEE, S. & MUN, C. W. 2013. Evaluation of the Effectiveness of Pregabalin in Alleviating Pain Associated with Fibromyalgia: Using Functional Magnetic Resonance Imaging Study. *Plos One,* 8.

KOBAYASHI, Y., KURATA, J., SEKIGUCHI, M., KOKUBUN, M., AKAISHIZAWA, T., CHIBA, Y., KONNO, S. & KIKUCHI, S. 2009. Augmented cerebral activation by lumbar mechanical stimulus in chronic low back pain patients: an FMRI study. *Spine (Phila Pa 1976),* 34**,** 2431-6.

KONG, J., JENSEN, K., LOIOTILE, R., CHEETHAM, A., WEY, H. Y., TAN, Y., ROSEN, B., SMOLLER, J. W., KAPTCHUK, T. J. & GOLLUB, R. L. 2013. Functional connectivity of the frontoparietal network predicts cognitive modulation of pain. *Pain,* 154**,** 459-67.

KONG, J., LOGGIA, M. L., ZYLONEY, C., TU, P., LAVIOLETTE, P. & GOLLUB, R. L. 2010. Exploring the brain in pain: activations, deactivations and their relation. *Pain,* 148**,** 257-67.

KONG, J., WHITE, N. S., KWONG, K. K., VANGEL, M. G., ROSMAN, I. S., GRACELY, R. H. & GOLLUB, R. L. 2006. Using fMRI to dissociate sensory encoding from cognitive evaluation of heat pain intensity. *Hum Brain Mapp,* 27**,** 715-21.

KOYAMA, T., MCHAFFIE, J. G., LAURIENTI, P. J. & COGHILL, R. C. 2005. The subjective experience of pain: where expectations become reality. *Proc Natl Acad Sci U S A,* 102**,** 12950-5.

KUCYI, A., SALOMONS, T. V. & DAVIS, K. D. 2013. Mind wandering away from pain dynamically engages antinociceptive and default mode brain networks. *Proc Natl Acad Sci U S A,* 110**,** 18692-7.

KURATA, J., THULBORN, K. R. & FIRESTONE, L. L. 2005. The cross-modal interaction between pain-related and saccade-related cerebral activation: a preliminary study by event-related functional magnetic resonance imaging. *Anesth Analg,* 101**,** 449-56, table of contents.

KURATA, J., THULBORN, K. R., GYULAI, F. E. & FIRESTONE, L. L. 2002. Early decay of pain-related cerebral activation in functional magnetic resonance imaging: comparison with visual and motor tasks. *Anesthesiology,* 96**,** 35-44.

LA CESA, S., TINELLI, E., TOSCHI, N., DI STEFANO, G., COLLORONE, S., ACETI, A., FRANCIA, A., CRUCCU, G., TRUINI, A. & CARAMIA, F. 2014. fMRI pain activation in the periaqueductal gray in healthy volunteers during the cold pressor test. *Magn Reson Imaging,* 32**,** 236-40.

LANDGREBE, M., BARTA, W., ROSENGARTH, K., FRICK, U., HAUSER, S., LANGGUTH, B., RUTSCHMANN, R., GREENLEE, M. W., HAJAK, G. & EICHHAMMER, P. 2008. Neuronal correlates of symptom formation in functional somatic syndromes: a fMRI study. *Neuroimage,* 41**,** 1336-44.

LEBEL, A., BECERRA, L., WALLIN, D., MOULTON, E. A., MORRIS, S., PENDSE, G., JASCIEWICZ, J., STEIN, M., AIELLO-LAMMENS, M., GRANT, E., BERDE, C. & BORSOOK, D. 2008. fMRI reveals distinct CNS processing during symptomatic and recovered complex regional pain syndrome in children. *Brain,* 131**,** 1854-79.

LEE, M. C., ZAMBREANU, L., MENON, D. K. & TRACEY, I. 2008. Identifying brain activity specifically related to the maintenance and perceptual consequence of central sensitization in humans. *J Neurosci,* 28**,** 11642-9.

LILJENCRANTZ, J., BJORNSDOTTER, M., MORRISON, I., BERGSTRAND, S., CEKO, M., SEMINOWICZ, D. A., COLE, J., BUSHNELL, M. C. & OLAUSSON, H. 2013. Altered C-tactile processing in human dynamic tactile allodynia. *Pain,* 154**,** 227-34.

LLOYD, D., FINDLAY, G., ROBERTS, N. & NURMIKKO, T. 2008. Differences in low back pain behavior are reflected in the cerebral response to tactile stimulation of the lower back. *Spine (Phila Pa 1976),* 33**,** 1372-7.

LLOYD, D. M., FINDLAY, G., ROBERTS, N. & NURMIKKO, T. 2014. Illness behavior in patients with chronic low back pain and activation of the affective circuitry of the brain. *Psychosom Med,* 76**,** 413-21.

LOPEZ-SOLA, M., PUJOL, J., HERNANDEZ-RIBAS, R., HARRISON, B. J., CONTRERAS-RODRIGUEZ, O., SORIANO-MAS, C., DEUS, J., ORTIZ, H., MENCHON, J. M., VALLEJO, J. & CARDONER, N. 2010a. Effects of duloxetine treatment on brain response to painful stimulation in major depressive disorder. *Neuropsychopharmacology,* 35**,** 2305-17.

LOPEZ-SOLA, M., PUJOL, J., HERNANDEZ-RIBAS, R., HARRISON, B. J., ORTIZ, H., SORIANO-MAS, C., DEUS, J., MENCHON, J. M., VALLEJO, J. & CARDONER, N. 2010b. Dynamic assessment of the right lateral frontal cortex response to painful stimulation. *Neuroimage,* 50**,** 1177-87.

LUI, F., DUZZI, D., CORRADINI, M., SERAFINI, M., BARALDI, P. & PORRO, C. A. 2008. Touch or pain? Spatio-temporal patterns of cortical fMRI activity following brief mechanical stimuli. *Pain,* 138**,** 362-74.

LUTZ, A., MCFARLIN, D. R., PERLMAN, D. M., SALOMONS, T. V. & DAVIDSON, R. J. 2013. Altered anterior insula activation during anticipation and experience of painful stimuli in expert meditators. *Neuroimage,* 64**,** 538-46.

MAEDA, L., ONO, M., KOYAMA, T., OSHIRO, Y., SUMITANI, M., MASHIMO, T. & SHIBATA, M. 2011. Human brain activity associated with painful mechanical stimulation to muscle and bone. *J Anesth,* 25**,** 523-30.

MAIHOFNER, C., FORSTER, C., BIRKLEIN, F., NEUNDORFER, B. & HANDWERKER, H. O. 2005. Brain processing during mechanical hyperalgesia in complex regional pain syndrome: a functional MRI study. *Pain,* 114**,** 93-103.

MAIHOFNER, C. & HANDWERKER, H. O. 2005. Differential coding of hyperalgesia in the human brain: a functional MRI study. *Neuroimage,* 28**,** 996-1006.

MAIHOFNER, C., HANDWERKER, H. O. & BIRKLEIN, F. 2006a. Functional imaging of allodynia in complex regional pain syndrome. *Neurology,* 66**,** 711-7.

MAIHOFNER, C., HERZNER, B. & OTTO HANDWERKER, H. 2006b. Secondary somatosensory cortex is important for the sensory-discriminative dimension of pain: a functional MRI study. *Eur J Neurosci,* 23**,** 1377-83.

MAIHOFNER, C., SCHMELZ, M., FORSTER, C., NEUNDORFER, B. & HANDWERKER, H. O. 2004. Neural activation during experimental allodynia: a functional magnetic resonance imaging study. *Eur J Neurosci,* 19**,** 3211-8.

MAIHOFNER, C., SEIFERT, F. & DECOL, R. 2011. Activation of central sympathetic networks during innocuous and noxious somatosensory stimulation. *Neuroimage,* 55**,** 216-24.

MANTINI, D., CAULO, M., FERRETTI, A., ROMANI, G. L. & TARTARO, A. 2009. Noxious somatosensory stimulation affects the default mode of brain function: evidence from functional MR imaging. *Radiology,* 253**,** 797-804.

MARKL, A., YU, T., VOGEL, D., MULLER, F., KOTCHOUBEY, B. & LANG, S. 2013. Brain processing of pain in patients with unresponsive wakefulness syndrome. *Brain Behav,* 3**,** 95-103.

MARTIN, L., BORCKARDT, J. J., REEVES, S. T., FROHMAN, H., BEAM, W., NAHAS, Z., JOHNSON, K., YOUNGER, J., MADAN, A., PATTERSON, D. & GEORGE, M. 2013. A pilot functional MRI study of the effects of prefrontal rTMS on pain perception. *Pain medicine (Malden, Mass ),* 14**,** 999-1009.

MISRA, G. & COOMBES, S. A. 2014. Neuroimaging Evidence of Motor Control and Pain Processing in the Human Midcingulate Cortex. *Cereb Cortex*.

MOBASCHER, A., BRINKMEYER, J., THIELE, H., TOLIAT, M. R., STEFFENS, M., WARBRICK, T., MUSSO, F., WITTSACK, H. J., SALEH, A., SCHNITZLER, A. & WINTERER, G. 2010a. The val158met polymorphism of human catechol-O-methyltransferase (COMT) affects anterior cingulate cortex activation in response to painful laser stimulation. *Mol Pain,* 6**,** 32.

MOBASCHER, A., BRINKMEYER, J., WARBRICK, T., MUSSO, F., SCHLEMPER, V., WITTSACK, H. J., SALEH, A., SCHNITZLER, A. & WINTERER, G. 2010b. Brain activation patterns underlying fast habituation to painful laser stimuli. *Int J Psychophysiol,* 75**,** 16-24.

MOBASCHER, A., BRINKMEYER, J., WARBRICK, T., MUSSO, F., WITTSACK, H. J., SALEH, A., SCHNITZLER, A. & WINTERER, G. 2009a. Laser-evoked potential P2 single-trial amplitudes covary with the fMRI BOLD response in the medial pain system and interconnected subcortical structures. *Neuroimage,* 45**,** 917-26.

MOBASCHER, A., BRINKMEYER, J., WARBRICK, T., MUSSO, F., WITTSACK, H. J., STOERMER, R., SALEH, A., SCHNITZLER, A. & WINTERER, G. 2009b. Fluctuations in electrodermal activity reveal variations in single trial brain responses to painful laser stimuli--a fMRI/EEG study. *Neuroimage,* 44**,** 1081-92.

MOCHIZUKI, H., SADATO, N., SAITO, D. N., TOYODA, H., TASHIRO, M., OKAMURA, N. & YANAI, K. 2007. Neural correlates of perceptual difference between itching and pain: a human fMRI study. *Neuroimage,* 36**,** 706-17.

MOHR, C., LEYENDECKER, S., MANGELS, I., MACHNER, B., SANDER, T. & HELMCHEN, C. 2008. Central representation of cold-evoked pain relief in capsaicin induced pain: an event-related fMRI study. *Pain,* 139**,** 416-30.

MORRISON, I. & DOWNING, P. E. 2007. Organization of felt and seen pain responses in anterior cingulate cortex. *Neuroimage,* 37**,** 642-51.

MORRISON, I., LLOYD, D., DI PELLEGRINO, G. & ROBERTS, N. 2004. Vicarious responses to pain in anterior cingulate cortex: is empathy a multisensory issue? *Cogn Affect Behav Neurosci,* 4**,** 270-8.

MOULTON, E. A., BECERRA, L., MALEKI, N., PENDSE, G., TULLY, S., HARGREAVES, R., BURSTEIN, R. & BORSOOK, D. 2011. Painful heat reveals hyperexcitability of the temporal pole in interictal and ictal migraine States. *Cereb Cortex,* 21**,** 435-48.

MOULTON, E. A., KEASER, M. L., GULLAPALLI, R. P. & GREENSPAN, J. D. 2005. Regional intensive and temporal patterns of functional MRI activation distinguishing noxious and innocuous contact heat. *J Neurophysiol,* 93**,** 2183-93.

MOULTON, E. A., PENDSE, G., BECERRA, L. R. & BORSOOK, D. 2012. BOLD responses in somatosensory cortices better reflect heat sensation than pain. *J Neurosci,* 32**,** 6024-31.

MOULTON, E. A., PENDSE, G., MORRIS, S., STRASSMAN, A., AIELLO-LAMMENS, M., BECERRA, L. & BORSOOK, D. 2007. Capsaicin-induced thermal hyperalgesia and sensitization in the human trigeminal nociceptive pathway: an fMRI study. *Neuroimage,* 35**,** 1586-600.

NAGLATZKI, R. P., SCHLAMANN, M., GASSER, T., LADD, M. E., SURE, U., FORSTING, M. & GIZEWSKI, E. R. 2012. Cerebral somatic pain modulation during autogenic training in fMRI. *Eur J Pain,* 16**,** 1293-301.

NICKEL, F. T., OTT, S., MOEHRINGER, S., SAAKE, M., DOERFLER, A., SEIFERT, F. & MAIHOEFNER, C. 2014. Brain correlates of short-term habituation to repetitive electrical noxious stimulation. *European Journal of Pain,* 18**,** 56-66.

NICOTRA, A., CRITCHLEY, H. D., MATHIAS, C. J. & DOLAN, R. J. 2006. Emotional and autonomic consequences of spinal cord injury explored using functional brain imaging. *Brain,* 129**,** 718-28.

OBERMANN, M., PLEGER, B., DE GREIFF, A., STUDE, P., KAUBE, H., DIENER, H. C. & KATSARAVA, Z. 2009. Temporal summation of trigeminal pain in human anterior cingulate cortex. *Neuroimage,* 46**,** 193-200.

OCHSNER, K. N., LUDLOW, D. H., KNIERIM, K., HANELIN, J., RAMACHANDRAN, T., GLOVER, G. C. & MACKEY, S. C. 2006. Neural correlates of individual differences in pain-related fear and anxiety. *Pain,* 120**,** 69-77.

OSHIRO, Y., QUEVEDO, A. S., MCHAFFIE, J. G., KRAFT, R. A. & COGHILL, R. C. 2007. Brain mechanisms supporting spatial discrimination of pain. *J Neurosci,* 27**,** 3388-94.

OSHIRO, Y., QUEVEDO, A. S., MCHAFFIE, J. G., KRAFT, R. A. & COGHILL, R. C. 2009. Brain mechanisms supporting discrimination of sensory features of pain: a new model. *J Neurosci,* 29**,** 14924-31.

PARKS, E. L., GEHA, P. Y., BALIKI, M. N., KATZ, J., SCHNITZER, T. J. & APKARIAN, A. V. 2011. Brain activity for chronic knee osteoarthritis: dissociating evoked pain from spontaneous pain. *Eur J Pain,* 15**,** 843 e1-14.

PELTZ, E., SEIFERT, F., DECOL, R., DORFLER, A., SCHWAB, S. & MAIHOFNER, C. 2011. Functional connectivity of the human insular cortex during noxious and innocuous thermal stimulation. *Neuroimage,* 54**,** 1324-35.

PERINI, I., BERGSTRAND, S. & MORRISON, I. 2013. Where pain meets action in the human brain. *J Neurosci,* 33**,** 15930-9.

PEYRON, R., SCHNEIDER, F., FAILLENOT, I., CONVERS, P., BARRAL, F. G., GARCIA-LARREA, L. & LAURENT, B. 2004. An fMRI study of cortical representation of mechanical allodynia in patients with neuropathic pain. *Neurology,* 63**,** 1838-46.

PICHE, M., ARSENAULT, M. & RAINVILLE, P. 2010. Dissection of perceptual, motor and autonomic components of brain activity evoked by noxious stimulation. *Pain,* 149**,** 453-62.

PLONER, M., LEE, M. C., WIECH, K., BINGEL, U. & TRACEY, I. 2010. Prestimulus functional connectivity determines pain perception in humans. *Proc Natl Acad Sci U S A,* 107**,** 355-60.

POMARES, F. B., FAILLENOT, I., BARRAL, F. G. & PEYRON, R. 2013. The 'where' and the 'when' of the BOLD response to pain in the insular cortex. Discussion on amplitudes and latencies. *NeuroImage,* 64**,** 466-75.

PUJOL, J., LOPEZ-SOLA, M., ORTIZ, H., VILANOVA, J. C., HARRISON, B. J., YUCEL, M., SORIANO-MAS, C., CARDONER, N. & DEUS, J. 2009. Mapping brain response to pain in fibromyalgia patients using temporal analysis of FMRI. *PLoS One,* 4**,** e5224.

PUKALL, C. F., STRIGO, I. A., BINIK, Y. M., AMSEL, R., KHALIFE, S. & BUSHNELL, M. C. 2005. Neural correlates of painful genital touch in women with vulvar vestibulitis syndrome. *Pain,* 115**,** 118-27.

QIU, Y., NOGUCHI, Y., HONDA, M., NAKATA, H., TAMURA, Y., TANAKA, S., SADATO, N., WANG, X., INUI, K. & KAKIGI, R. 2006. Brain processing of the signals ascending through unmyelinated C fibers in humans: an event-related functional magnetic resonance imaging study. *Cereb Cortex,* 16**,** 1289-95.

RAIJ, T. T., NUMMINEN, J., NARVANEN, S., HILTUNEN, J. & HARI, R. 2005. Brain correlates of subjective reality of physically and psychologically induced pain. *Proc Natl Acad Sci U S A,* 102**,** 2147-51.

REMY, F., FRANKENSTEIN, U. N., MINCIC, A., TOMANEK, B. & STROMAN, P. W. 2003. Pain modulates cerebral activity during cognitive performance. *Neuroimage,* 19**,** 655-64.

ROBERTS, K., PAPADAKI, A., GONCALVES, C., TIGHE, M., ATHERTON, D., SHENOY, R., MCROBBIE, D. & ANAND, P. 2008. Contact Heat Evoked Potentials Using Simultaneous Eeg And Fmri And Their Correlation With Evoked Pain. *BMC Anesthesiol,* 8**,** 8.

ROLLS, E. T., O'DOHERTY, J., KRINGELBACH, M. L., FRANCIS, S., BOWTELL, R. & MCGLONE, F. 2003. Representations of pleasant and painful touch in the human orbitofrontal and cingulate cortices. *Cereb Cortex,* 13**,** 308-17.

ROY, M., PICHE, M., CHEN, J.-I., PERETZ, I. & RAINVILLE, P. 2009. Cerebral and spinal modulation of pain by emotions. *Proceedings of the National Academy of Sciences of the United States of America,* 106**,** 20900-5.

RUSSO, A., TESSITORE, A., ESPOSITO, F., MARCUCCIO, L., GIORDANO, A., CONFORTI, R., TRUINI, A., PACCONE, A., D'ONOFRIO, F. & TEDESCHI, G. 2012. Pain processing in patients with migraine: an event-related fMRI study during trigeminal nociceptive stimulation. *J Neurol,* 259**,** 1903-12.

SCHOEDEL, A. L., ZIMMERMANN, K., HANDWERKER, H. O. & FORSTER, C. 2008. The influence of simultaneous ratings on cortical BOLD effects during painful and non-painful stimulation. *Pain,* 135**,** 131-41.

SCHWEINHARDT, P., GLYNN, C., BROOKS, J., MCQUAY, H., JACK, T., CHESSELL, I., BOUNTRA, C. & TRACEY, I. 2006. An fMRI study of cerebral processing of brush-evoked allodynia in neuropathic pain patients. *Neuroimage,* 32**,** 256-65.

SEIFERT, F., BSCHORER, K., DE COL, R., FILITZ, J., PELTZ, E., KOPPERT, W. & MAIHOFNER, C. 2009. Medial prefrontal cortex activity is predictive for hyperalgesia and pharmacological antihyperalgesia. *J Neurosci,* 29**,** 6167-75.

SEIFERT, F., JUNGFER, I., SCHMELZ, M. & MAIHOFNER, C. 2008. Representation of UV-B-induced thermal and mechanical hyperalgesia in the human brain: a functional MRI study. *Hum Brain Mapp,* 29**,** 1327-42.

SEIFERT, F. & MAIHOFNER, C. 2007. Representation of cold allodynia in the human brain--a functional MRI study. *Neuroimage,* 35**,** 1168-80.

SEIFERT, F., SCHUBERTH, N., DE COL, R., PELTZ, E., NICKEL, F. T. & MAIHOFNER, C. 2013. Brain activity during sympathetic response in anticipation and experience of pain. *Human brain mapping,* 34**,** 1768-82.

SEMINOWICZ, D. A. & DAVIS, K. D. 2006. Cortical responses to pain in healthy individuals depends on pain catastrophizing. *Pain,* 120**,** 297-306.

SHELTON, L., PENDSE, G., MALEKI, N., MOULTON, E. A., LEBEL, A., BECERRA, L. & BORSOOK, D. 2012. Mapping pain activation and connectivity of the human habenula. *J Neurophysiol,* 107**,** 2633-48.

SHENOY, R., ROBERTS, K., PAPADAKI, A., MCROBBIE, D., TIMMERS, M., MEERT, T. & ANAND, P. 2011. Functional MRI brain imaging studies using the Contact Heat Evoked Potential Stimulator (CHEPS) in a human volunteer topical capsaicin pain model. *J Pain Res,* 4**,** 365-71.

SHUKLA, S., TOROSSIAN, A., DUANN, J. R. & LEUNG, A. 2011. The analgesic effect of electroacupuncture on acute thermal pain perception--a central neural correlate study with fMRI. *Mol Pain,* 7**,** 45.

SMITH, K. A., PLOGHAUS, A., COWEN, P. J., MCCLEERY, J. M., GOODWIN, G. M., SMITH, S., TRACEY, I. & MATTHEWS, P. M. 2002. Cerebellar responses during anticipation of noxious stimuli in subjects recovered from depression. Functional magnetic resonance imaging study. *Br J Psychiatry,* 181**,** 411-5.

SOFINA, T., KAMIL, W. A. & AHMAD, A. H. 2014. fMRI of pain studies using laser-induced heat on skin with and without the loved one near the subject - a pilot study on 'love hurts'. *9th National Seminar on Medical Physics (Nsmp2014),* 546.

SPRENGER, C., BINGEL, U. & BUCHEL, C. 2011. Treating pain with pain: supraspinal mechanisms of endogenous analgesia elicited by heterotopic noxious conditioning stimulation. *Pain,* 152**,** 428-39.

STAMMLER, T., DE COL, R., SEIFERT, F. & MAIHOFNER, C. 2008. Functional imaging of sensory decline and gain induced by differential noxious stimulation. *Neuroimage,* 42**,** 1151-63.

STARR, C. J., SAWAKI, L., WITTENBERG, G. F., BURDETTE, J. H., OSHIRO, Y., QUEVEDO, A. S. & COGHILL, R. C. 2009. Roles of the insular cortex in the modulation of pain: insights from brain lesions. *J Neurosci,* 29**,** 2684-94.

STRAUBE, T., SCHMIDT, S., WEISS, T., MENTZEL, H. J. & MILTNER, W. H. 2009. Sex differences in brain activation to anticipated and experienced pain in the medial prefrontal cortex. *Hum Brain Mapp,* 30**,** 689-98.

STRIGO, I. A., DUNCAN, G. H., BOIVIN, M. & BUSHNELL, M. C. 2003. Differentiation of visceral and cutaneous pain in the human brain. *J Neurophysiol,* 89**,** 3294-303.

SYMONDS, L. L., GORDON, N. S., BIXBY, J. C. & MANDE, M. M. 2006. Right-lateralized pain processing in the human cortex: an FMRI study. *J Neurophysiol,* 95**,** 3823-30.

TAKAHASHI, K., TAGUCHI, T., TANAKA, S., SADATO, N., QIU, Y., KAKIGI, R. & MIZUMURA, K. 2011. Painful muscle stimulation preferentially activates emotion-related brain regions compared to painful skin stimulation. *Neuroscience research,* 70**,** 285-93.

TER MINASSIAN, A., RICALENS, E., HUMBERT, S., DUC, F., AUBE, C. & BEYDON, L. 2013. Dissociating anticipation from perception: Acute pain activates default mode network. *Human brain mapping,* 34**,** 2228-43.

THEYSOHN, N., CHOI, K.-E., GIZEWSKI, E. R., WEN, M., RAMPP, T., GASSER, T., DOBOS, G. J., FORSTING, M. & MUSIAL, F. 2014. Acupuncture-Related Modulation of Pain-Associated Brain Networks During Electrical Pain Stimulation: A Functional Magnetic Resonance Imaging Study. *Journal of Alternative and Complementary Medicine,* 20**,** 893-900.

TRACEY, I., BECERRA, L., CHANG, I., BREITER, H., JENKINS, L., BORSOOK, D. & GONZALEZ, R. G. 2000. Noxious hot and cold stimulation produce common patterns of brain activation in humans: a functional magnetic resonance imaging study. *Neurosci Lett,* 288**,** 159-62.

TSENG, M.-T., CHIANG, M.-C., CHAO, C.-C., TSENG, W.-Y. I. & HSIEH, S.-T. 2013. fMRI evidence of degeneration-induced neuropathic pain in diabetes: enhanced limbic and striatal activations. *Human brain mapping,* 34**,** 2733-46.

TSENG, M. T., TSENG, W. Y., CHAO, C. C., LIN, H. E. & HSIEH, S. T. 2010. Distinct and shared cerebral activations in processing innocuous versus noxious contact heat revealed by functional magnetic resonance imaging. *Hum Brain Mapp,* 31**,** 743-57.

UEMATSU, H., SHIBATA, M., MIYAUCHI, S. & MASHIMO, T. 2011. Brain imaging of mechanically induced muscle versus cutaneous pain. *Neurosci Res,* 70**,** 78-84.

VACHON-PRESSEAU, E., ROY, M., MARTEL, M. O., CARON, E., MARIN, M. F., CHEN, J., ALBOUY, G., PLANTE, I., SULLIVAN, M. J., LUPIEN, S. J. & RAINVILLE, P. 2013. The stress model of chronic pain: evidence from basal cortisol and hippocampal structure and function in humans. *Brain,* 136**,** 815-27.

VALET, M., SPRENGER, T., BOECKER, H., WILLOCH, F., RUMMENY, E., CONRAD, B., ERHARD, P. & TOLLE, T. R. 2004. Distraction modulates connectivity of the cingulo-frontal cortex and the midbrain during pain--an fMRI analysis. *Pain,* 109**,** 399-408.

VANHAUDENHUYSE, A., BOLY, M., BALTEAU, E., SCHNAKERS, C., MOONEN, G., LUXEN, A., LAMY, M., DEGUELDRE, C., BRICHANT, J. F., MAQUET, P., LAUREYS, S. & FAYMONVILLE, M. E. 2009. Pain and non-pain processing during hypnosis: a thulium-YAG event-related fMRI study. *Neuroimage,* 47**,** 1047-54.

VARTIAINEN, N., KALLIO-LAINE, K., HLUSHCHUK, Y., KIRVESKARI, E., SEPPANEN, M., AUTTI, H., JOUSMAKI, V., FORSS, N., KALSO, E. & HARI, R. 2009. Changes in brain function and morphology in patients with recurring herpes simplex virus infections and chronic pain. *Pain,* 144**,** 200-8.

VELDHUIJZEN, D. S., NEMENOV, M. I., KEASER, M., ZHUO, J., GULLAPALLI, R. P. & GREENSPAN, J. D. 2009. Differential brain activation associated with laser-evoked burning and pricking pain: An event-related fMRI study. *Pain,* 141**,** 104-13.

VILLEMURE, C. & BUSHNELL, M. C. 2009. Mood influences supraspinal pain processing separately from attention. *J Neurosci,* 29**,** 705-15.

WAGER, T. D., RILLING, J. K., SMITH, E. E., SOKOLIK, A., CASEY, K. L., DAVIDSON, R. J., KOSSLYN, S. M., ROSE, R. M. & COHEN, J. D. 2004. Placebo-induced changes in FMRI in the anticipation and experience of pain. *Science,* 303**,** 1162-7.

WAGNER, G., KOSCHKE, M., LEUF, T., SCHLOSSER, R. & BAR, K. J. 2009. Reduced heat pain thresholds after sad-mood induction are associated with changes in thalamic activity. *Neuropsychologia,* 47**,** 980-7.

WANG, Y., WANG, J. Y. & LUO, F. 2011. Why self-induced pain feels less painful than externally generated pain: distinct brain activation patterns in self- and externally generated pain. *PLoS One,* 6**,** e23536.

WEY, H. Y., CATANA, C., HOOKER, J. M., DOUGHERTY, D. D., KNUDSEN, G. M., WANG, D. J. J., CHONDE, D. B., ROSEN, B. R., GOLLUB, R. L. & KONG, J. 2014. Simultaneous fMRI-PET of the opioidergic pain system in human brain. *Neuroimage,* 102**,** 275-282.

WIECH, K., KALISCH, R., WEISKOPF, N., PLEGER, B., STEPHAN, K. E. & DOLAN, R. J. 2006. Anterolateral prefrontal cortex mediates the analgesic effect of expected and perceived control over pain. *J Neurosci,* 26**,** 11501-9.

WIECH, K., LIN, C. S., BRODERSEN, K. H., BINGEL, U., PLONER, M. & TRACEY, I. 2010. Anterior insula integrates information about salience into perceptual decisions about pain. *J Neurosci,* 30**,** 16324-31.

ZAMBREANU, L., WISE, R. G., BROOKS, J. C. W., IANNETTI, G. D. & TRACEY, I. 2005. A role for the brainstem in central sensitisation in humans. Evidence from functional magnetic resonance imaging. *Pain,* 114**,** 397-407.

ZIV, M., TOMER, R., DEFRIN, R. & HENDLER, T. 2010. Individual sensitivity to pain expectancy is related to differential activation of the hippocampus and amygdala. *Human brain mapping,* 31**,** 326-38.
