# Supplementary material for: Functional reorganisation in chronic pain and neural correlates of pain sensitisation: A coordinate based meta-analysis of 266 cutaneous pain fMRI studies
Source: Neurosci Biobehav Rev. 2016 Sep;68:120–33. doi: 10.1016/j.neubiorev.2016.04.001 (PMC5554296; doi:10.1016/j.neubiorev.2016.04.001)
Supplement: Supplementary Table S2 [file mmc2.docx]

**Tables S2.1-7**

**Table S2.1. Significant clusters from experimental pain studies in healthy volunteers.**

| **Anatomical region (Brodmann Area)** | **Centroid Coordinates** | | | **P-value** | **Number of studies contributing0** |
| --- | --- | --- | --- | --- | --- |
|  | **X** | **Y** | **Z** |  |  |
| R Postcentral Gyrus (40) | 51.6 | -25.1 | 21.2 | 0 | 79 |
| R Insula (13) | 36.1 | 12.9 | 4.6 | 0 | 88 |
| L Claustrum | -32.4 | 14.4 | 7.0 | 0 | 70 |
| L Thalamus – Ventral Posterior Lateral Nucleus | -12.5 | -16.3 | 7.3 | 0 | 54 |
| R Cingulate Gyrus (24) | 2.1 | 4.3 | 40.0 | 0 | 59 |
| L Postcentral Gyrus (40) | -55.3 | -24.2 | 19.3 | 0 | 59 |
| L Insula (13) | -36.9 | 1.4 | 7.6 | 0 | 55 |
| L Cingulate Gyrus (32) | -0.1 | 17.8 | 35.2 | 0 | 48 |
| L Insula (13) | -37.8 | -17.6 | 13.1 | 0 | 38 |
| R Thalamus – Medial Dorsal Nucleus | 9.6 | -15.8 | 8.6 | 0 | 72 |
| R Claustrum | 33.3 | 0.0 | 11.3 | 0 | 39 |
| R Medial Frontal Gyrus (6) | 3.9 | 15.8 | 47.1 | 0 | 27 |
| R Lentiform Nucleus | 19.8 | 7.4 | 3.1 | 0 | 34 |
| R Insula (13) | 36.4 | -18.0 | 15.6 | 0 | 30 |
| L Inferior Parietal Lobule (40) | -56.1 | -34.7 | 23.0 | 0 | 32 |
| R Middle Frontal Gyrus (10) | 33.8 | 43.8 | 23.4 | 0.000003 | 21 |
| R Caudate | 10.2 | 6.7 | 9.8 | 0.000004 | 15 |
| R Parahippocampal Gyrus/Amygdala | 20.6 | -1.1 | -12.1 | 0.000004 | 12 |
| R Precentral Gyrus (44) | 49.4 | 0.4 | 9.1 | 0.000008 | 21 |
| R Thalamus | 8.8 | -1.6 | 5.3 | 0.000009 | 16 |
| L Cingulate Gyrus (23) | -1.2 | -28.3 | 27.2 | 0.000011 | 15 |
| L Cingulate Gyrus (24) | -7.5 | 1.4 | 35.6 | 0.000012 | 23 |
| L Precentral Gyrus (6) | -51.3 | -3.5 | 8.2 | 0.000034 | 19 |
| R Midbrain | 0.9 | -29.1 | -5.2 | 0.000037 | 12 |
| R Precentral Gyrus (44) | 50.2 | 11.0 | 6.3 | 0.000046 | 15 |
| L Inferior Parietal Lobule (40) | -42.0 | -40.4 | 39.2 | 0.000048 | 9 |
| R Inferior Frontal Gyrus (46) | 38.8 | 34.9 | 9.6 | 0.000055 | 15 |
| R Middle Frontal Gyrus (9) | 37.1 | 30.8 | 33.1 | 0.000063 | 8 |
| L Insula (13) | -47.4 | 4.1 | 4.2 | 0.000077 | 18 |
| R Inferior Parietal Lobule (40) | 51.1 | -42.8 | 31.7 | 0.000081 | 24 |
| L Lentiform Nucleus | -15.8 | 3.1 | 4.0 | 0.00011 | 15 |
| L Medial Frontal Gyrus (6) | -5.0 | 5.4 | 48.4 | 0.000114 | 19 |
| R Inferior Parietal Lobule (40) | 47.4 | -37.1 | 40.3 | 0.000127 | 15 |
| R Middle Frontal Gyrus (9) | 42.9 | 16.2 | 31.9 | 0.000139 | 9 |
| R Midbrain | 7.5 | -21.3 | -10.2 | 0.000151 | 13 |
| L Postcentral Gyrus (3) | -39.1 | -27.2 | 61.9 | 0.000377 | 7 |
| L Thalamus | -12.4 | -7.2 | 15.8 | 0.00052 | 12 |
| L Precentral Gyrus (6) | -47.8 | -0.9 | 31.2 | 0.000606 | 6 |
| R Middle Frontal Gyrus (9) | 33.3 | 36.7 | 26.4 | 0.000668 | 13 |
| R Inferior Parietal Lobule (40) | 40.5 | -52.3 | 46.4 | 0.000699 | 7 |
| L Midbrain | -7.0 | -17.6 | -9.5 | 0.000937 | 14 |
| R Culmen | 22.2 | -49.0 | -22.1 | 0.00096 | 9 |
| L Supramarginal Gyrus (40) | -55.0 | -39.9 | 32.9 | 0.001411 | 8 |
| L Insula (13) | -38.6 | -7.1 | 0.9 | 0.001472 | 8 |
| R Middle Frontal Gyrus (6) | 40.5 | 4.2 | 45.0 | 0.001489 | 6 |
| R Middle Frontal Gyrus (10) | 40.6 | 46.1 | 12.2 | 0.001579 | 10 |
| R Postcentral Gyrus (43) | 60.7 | -17.1 | 20.0 | 0.001591 | 7 |
| R Middle Frontal Gyrus (46) | 42.2 | 34.9 | 15.8 | 0.001591 | 12 |
| All clusters significant at FCDR p<0.05. Centroid coordinates reported in Talairach space. R – right, L – left. | | | | | |

**Table S2.2. Significant clusters from experimental pain studies in chronic pain cohorts.**

| **Anatomical region (Brodmann Area)** | **Centroid Coordinates** | | | **P-value** | **Number of studies contributing** |
| --- | --- | --- | --- | --- | --- |
|  | **X** | **Y** | **Z** |  |  |
| R Postcentral Gyrus (40) | 52.0 | -26.9 | 19.7 | 0 | 23 |
| R Precentral Gyrus (44) | 41.4 | 5.8 | 6.9 | 0 | 23 |
| L Postcentral Gyrus (40) | -53.4 | -25.0 | 20.6 | 0 | 21 |
| R Lentiform Nucleus | 23.6 | 4.2 | 3.1 | 0 | 11 |
| R Thalamus – Mammillary Body | 12.2 | -18.4 | 5.1 | 0 | 9 |
| R Insula (13) | 33.3 | 20.2 | 2.3 | 0.000002 | 15 |
| L Cingulate Gyrus (32) | 0.3 | 7.7 | 38.2 | 0.000012 | 18 |
| L Supramarginal Gyrus (40) | -41.8 | -49.4 | 35.1 | 0.000014 | 8 |
| L Claustrum | -35.0 | 2.5 | 5.4 | 0.000014 | 7 |
| L Insula (13) | -33.0 | 17.7 | 4.4 | 0.000057 | 16 |
| L Precentral Gyrus (6) | -46.7 | -3.2 | 4.8 | 0.000067 | 14 |
| L Lentiform Nucleus | -23.3 | -3.9 | 7.1 | 0.00007 | 9 |
| R Medial Frontal Gyrus (6) | 2.0 | -3.4 | 53.5 | 0.000084 | 11 |
| L Culmen | -27.8 | -56.5 | -21.7 | 0.002581 | 5 |
| L Thalamus | -10.4 | -8.3 | 9.8 | 0.002733 | 8 |
| All clusters significant at FCDR p<0.05. Centroid coordinates reported in Talairach space. R – right, L – left. | | | | | |

**Table S2.3. Significant clusters from CS group.**

| **Anatomical region (Brodmann Area)** | **Centroid Coordinates** | | | **P-value** | **Number of studies contributing** |
| --- | --- | --- | --- | --- | --- |
|  | **X** | **Y** | **Z** |  |  |
| R Insula (13) | 40.4 | 5.1 | 7.9 | 0 | 14 |
| L Postcentral Gyrus (40) | -54.4 | -26.1 | 20.6 | 0 | 13 |
| L Lentiform Nucleus | -22.6 | -7.0 | 8.7 | 0 | 12 |
| R Lentiform Nucleus | 21.6 | 4.8 | 4.8 | 0 | 7 |
| R Thalamus | 12.6 | -18.2 | 5.8 | 0.000002 | 6 |
| R Insula (13) | 50.5 | -28.5 | 20.8 | 0.000002 | 14 |
| L Cingulate Gyrus (32) | -0.2 | 9.2 | 38.4 | 0.000006 | 15 |
| R Insula (13) | 32.3 | 21.6 | 2.4 | 0.000012 | 8 |
| L Insula | -35.1 | 16.5 | 2.8 | 0.000022 | 11 |
| R Medial Frontal Gyrus (6) | 2.4 | -5.4 | 54.6 | 0.000741 | 7 |
| L Middle Frontal Gyrus (10) | -35.2 | 38.8 | 24.4 | 0.002319 | 7 |
| All clusters significant at FCDR p<0.05. Centroid coordinates reported in Talairach space. R – right, L – left. | | | | | |

**Table S2.4. Significant clusters from OS group.**

| **Anatomical region (Brodmann Area)** | **Centroid Coordinates** | | | **P-value** | **Number of studies contributing** |
| --- | --- | --- | --- | --- | --- |
|  | **X** | **Y** | **Z** |  |  |
| L Insula (13) | -45.3 | -2.9 | 5.7 | 0.000006 | 10 |
| R Insula (13) | 37.6 | 14.1 | 3.7 | 0.000084 | 12 |
| L Postcentral Gyrus (40) | -52.1 | -25.2 | 22.2 | 0.000113 | 8 |
| R Inferior Parietal Lobule (40) | 56.1 | -31.1 | 22.7 | 0.000132 | 5 |
| R Precentral Gyrus (13) | 49.2 | -14.1 | 13.4 | 0.000589 | 6 |
| L Insula (13) | -34.9 | 17.2 | 6.2 | 0.002576 | 7 |
| All clusters significant at FCDR p<0.05. Centroid coordinates reported in Talairach space. R – right, L – left. | | | | | |

**Table S2.5. Significant clusters from CS vs. OS comparison.**

| **Anatomical region (Brodmann Area)** | **Centroid Coordinates** | | | **P-value** | **Number of studies contributing** |
| --- | --- | --- | --- | --- | --- |
|  | **X** | **Y** | **Z** |  |  |
| **CS > OS** |  |  |  |  |  |
| L Lentiform Nucleus | -22.4 | -5.3 | 9.3 | 0.0016 | 10 |
| R Insula (13) | 45.0 | -32.6 | 19.5 | 0.0019 | 10 |
| L Middle Frontal Gyrus (9) | -32.7 | 40.9 | 26.8 | 0.0021 | 7 |
| R Insula (13) | 40.1 | 4.5 | 10.3 | 0.0041 | 11 |
| **OS > CS** |  |  |  |  |  |
| L Precentral Gyrus (43) | -49.7 | -7.1 | 9.9 | 0.001 | 10 |
| All clusters significant at FCDR p<0.1. Centroid coordinates reported in Talairach space. R – right, L – left. | | | | | |

**Table S2.6. Significant clusters from hyperalgesia group.**

| **Anatomical region (Brodmann Area)** | **Centroid Coordinates** | | | **P-value** | **Number of studies contributing** |
| --- | --- | --- | --- | --- | --- |
|  | **X** | **Y** | **Z** |  |  |
| L Insula (13) | -48.0 | -24.2 | 21.4 | 0 | 7 |
| R Insula (13) | 48.0 | -23.9 | 21.0 | 0 | 7 |
| R Insula (13) | 39.1 | 10.1 | 11.7 | 0.000001 | 9 |
| R Cingulate Gyrus (32) | 1.5 | 17.6 | 35.1 | 0.000013 | 8 |
| L Insula (13) | -36.3 | 16.8 | 10.7 | 0.000021 | 8 |
| L Thalamus | -10.7 | -15.0 | 6.1 | 0.000241 | 4 |
| R Superior Frontal Gyrus (8) | 17.9 | 45.7 | 35.7 | 0.00209 | 4 |
| L Lentiform Nucleus | -14.7 | 4.7 | 9.9 | 0.003973 | 4 |
| All clusters significant at FCDR p<0.05. Centroid coordinates reported in Talairach space. R – right, L – left. | | | | | |

**Table S2.7. Significant Clusters from the MAC Hyperalgesia v normalgesia.**

| **Anatomical region (Brodmann Area)** | **Centroid Coordinates** | | | **P-value** | **Number of studies contributing** |
| --- | --- | --- | --- | --- | --- |
|  | **X** | **Y** | **Z** |  |  |
| R Insula (13) | 38.7 | 9.8 | 7.8 | 0.000009 | 6 |
| L Insula (40) | -48.5 | -20.1 | 14.0 | 0.000396 | 7 |
| R Middle Frontal Gyrus (9) | 31.8 | 37.1 | 27.7 | 0.000526 | 4 |
| L Lentiform Nucleus | -14.4 | 5.8 | 6.5 | 0.000747 | 4 |
| L Cingulate Gyrus (32) | 0.5 | 17.9 | 32.0 | 0.001317 | 5 |
| R Inferior Parietal Lobule (40) | 41.3 | -45.2 | 40.9 | 0.002026 | 4 |
| L Insula (13) | -38.3 | 12.1 | 10.4 | 0.002065 | 6 |
| All clusters significant at FCDR p<0.05. Centroid coordinates reported in Talairach space. R – right, L – left. | | | | | |
