# Supplementary figures and images for: Functional reorganisation in chronic pain and neural correlates of pain sensitisation: A coordinate based meta-analysis of 266 cutaneous pain fMRI studies
Source: Neurosci Biobehav Rev. 2016 Sep;68:120–33. doi: 10.1016/j.neubiorev.2016.04.001 (PMC5554296; doi:10.1016/j.neubiorev.2016.04.001)

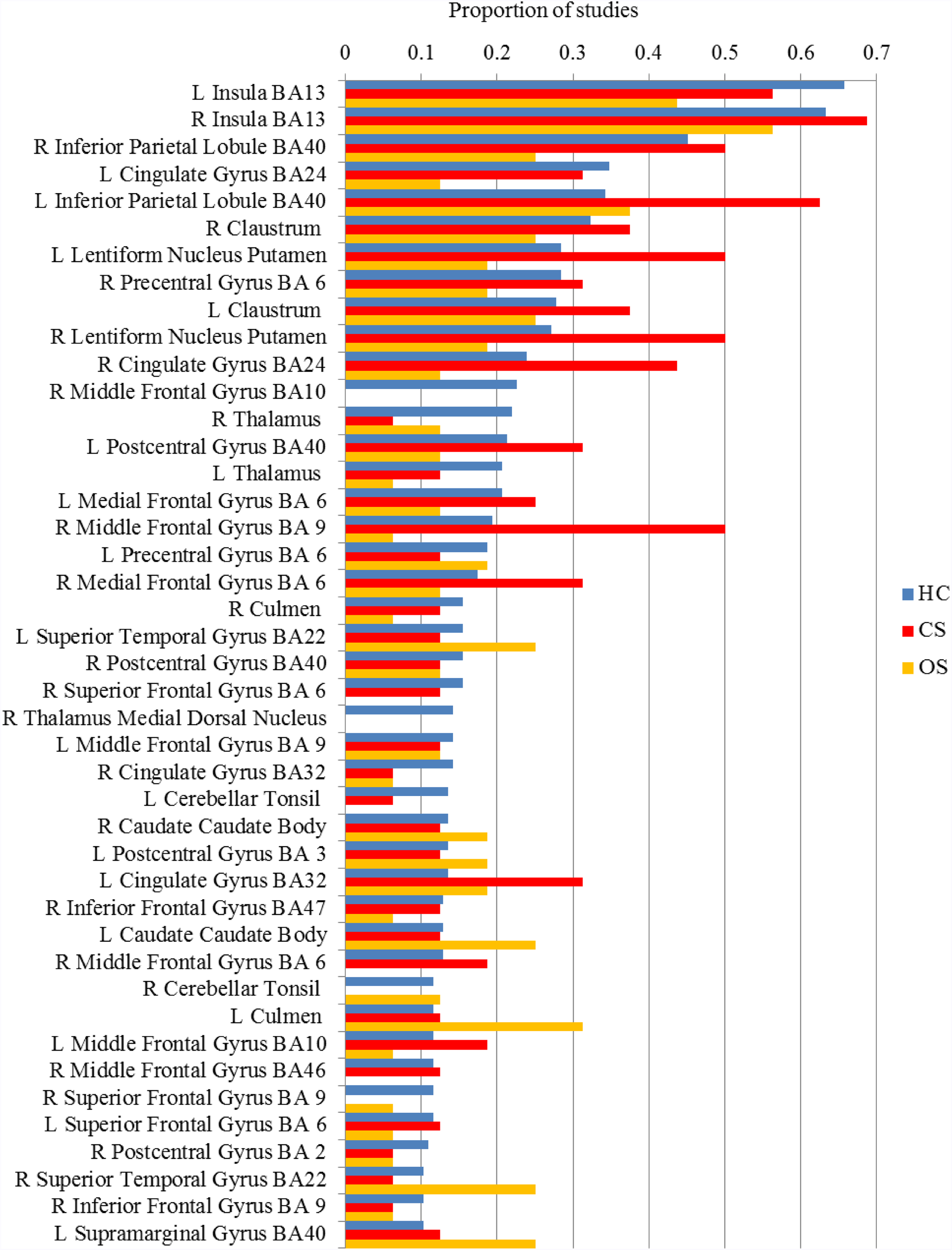

Supplement: Supplementary Fig. S1 — Most frequently reported structures in the CP sub-groups (red-CSCP, yellow-OSCP). HC (blue) are added for comparison. Structures reported at least by 10% of the studies in one group are displayed. [file mmc4.jpg]

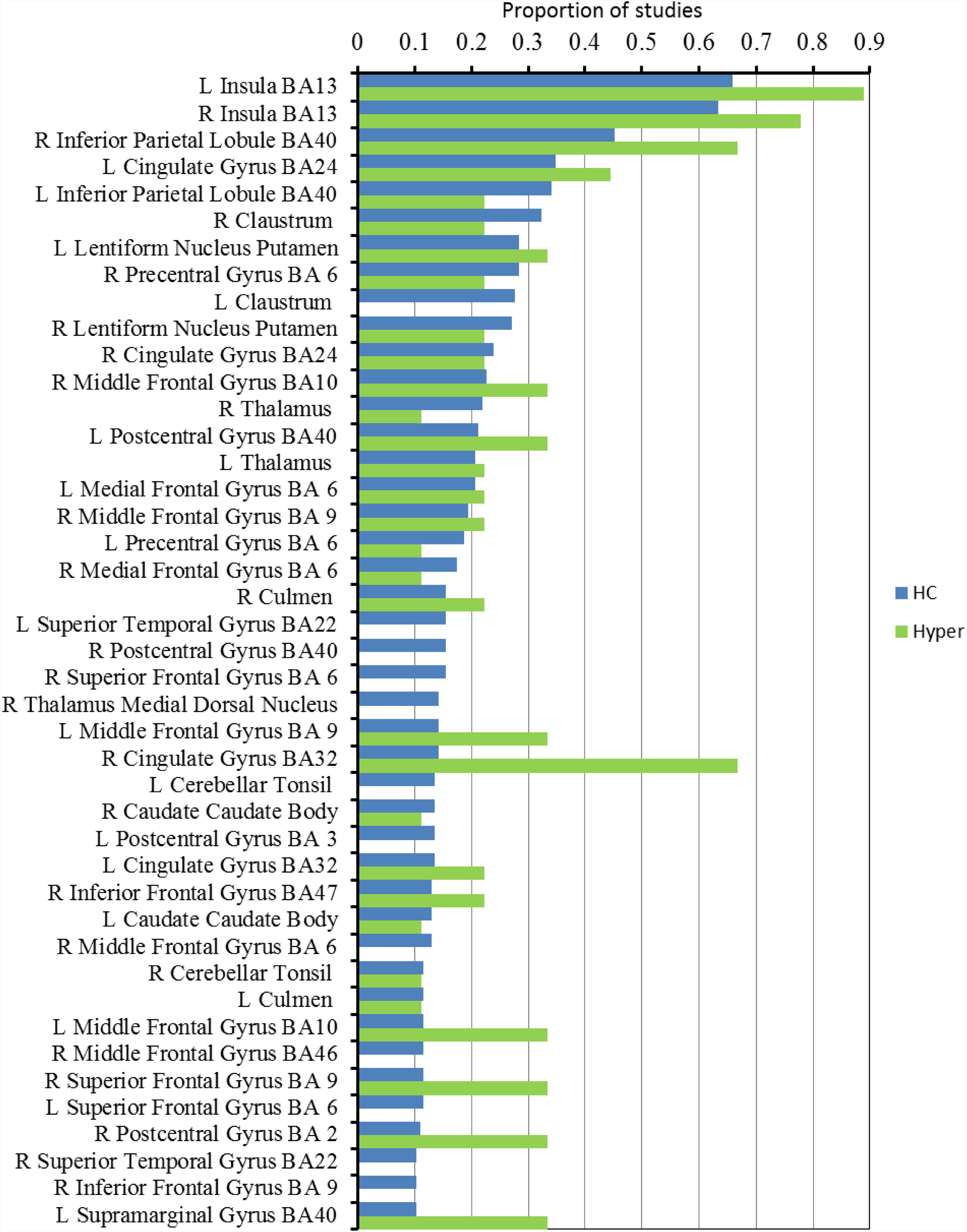

Supplement: Supplementary Fig. S2 — Frequency of reported structures in Hyperalgesia (green) and HC (blue) groups. Structures reported at least by 10% of the studies in one group are displayed. [file mmc5.jpg]
